# Enhancing the Efficacy of Active Pharmaceutical Ingredients in Medicinal Plants through Nanoformulations: A Promising Field

**DOI:** 10.3390/nano14191598

**Published:** 2024-10-03

**Authors:** Yuhao Chen, Yuying Tang, Yuanbo Li, Yukui Rui, Peng Zhang

**Affiliations:** 1 Beijing Key Laboratory of Farmland Soil Pollution Prevention and Remediation, College of Resources and Environmental Sciences, China Agricultural University, Beijing 100093, China; 18855380430@163.com (Y.C.); xtang12@126.com (Y.T.); liyuanbo@cau.edu.cn (Y.L.); 2 Tangshan Jinhai New Material Co., Ltd., Tangshan 063000, China; 3 Faculty of Resources and Environment, China Agricultural University, Shanghe County Baiqiao Town Science and Technology Courtyard, Jinan 250100, China; 4 Department of Environmental Science and Engineering, University of Science and Technology of China, Hefei 230026, China

**Keywords:** nanoformulations, medicinal plant, bioavailability, triggered release, targeting

## Abstract

This article explores the emerging field of nanomedicine as a drug delivery system, aimed at enhancing the therapeutic efficacy of active pharmaceutical ingredients in medicinal plants. The traditional methods of applying medicinal plants present several limitations, such as low bioavailability, poor solubility, challenges in accurately controlling drug dosage, and inadequate targeting. Nanoformulations represent an innovative approach in drug preparation that employs nanotechnology to produce nanoscale particles or carriers, which are designed to overcome these limitations. Nanoformulations offer distinct advantages, significantly enhancing the solubility and bioavailability of drugs, particularly for the poorly soluble components of medicinal plants. These formulations effectively enhance solubility, thereby facilitating better absorption and utilization by the human body, which in turn improves drug efficacy. Furthermore, nanomedicine enables targeted drug delivery, ensuring precise administration to the lesion site and minimizing side effects on healthy tissues. Additionally, nanoformulations can regulate drug release rates, extend the duration of therapeutic action, and enhance the stability of treatment effects. However, nanoformulations present certain limitations and potential risks; their stability and safety require further investigation, particularly regarding the potential toxicity with long-term use. Nevertheless, nanomaterials demonstrate substantial potential in augmenting the efficacy of active pharmaceutical ingredients in medicinal plants, offering novel approaches and methodologies for their development and application.

## 1. Introduction

The global healthcare field is experiencing an increasing demand for more effective, safe, and sustainable treatment methods [1]. Medicinal plants, a crucial component of traditional medicine, have a long history of use and considerable therapeutic potential. The World Health Organization reports that 80% of individuals in developing countries depend on natural medicines to meet their daily medical needs [2]. Approximately 11% of drugs listed in the World Health Organization’s Essential Medicines List are derived entirely from medicinal plants [2]. The global market for plant-based medicines is substantial, expanding at an annual rate of 13%; by 2050, market demand is projected to reach $5 trillion [3,4].

Medicinal plants refer to plants used for disease prevention and treatment, all or part of which can be used for medicinal purposes or as raw materials for the pharmaceutical industry [5]. With a total of 11,146 species of medicinal plants, China is one of the countries with the richest resources of medicinal plants. Additionally, China is the world’s largest producer and supplier of herbal medicines, with a long history of discovering, utilizing, and cultivating medicinal plants [6,7]. However, the share of Chinese herbal medicine in the global traditional medicine market is relatively small, representing only approximately 10% [8]. Low bioavailability and poor solvent solubility significantly impact the effectiveness and utilization of medicinal plants, contributing to their limited market share [9]. Many bioactive compounds in medicinal plants, including flavonoids, terpenes, and saponins, have high water solubility but exhibit low absorption by the human body. Due to their high molecular weight, which hinders the penetration of lipid membranes, their absorption rate is low, leading to reduced bioavailability and efficacy [10,11,12]. Furthermore, some valuable medicinal plant resources are scarce, and traditional preparation methods, such as decoctions, pills, ointments, etc., have led to the suboptimal utilization of their medicinal effects, resulting in resource waste [13]. For example, luteolin is a flavonoid compound found naturally in a variety of plants, fruits, and vegetables. In traditional Chinese medicine, luteolin is used to treat high blood pressure, cancer, and inflammatory diseases. However, its clinical application is severely limited due to its low solubility in water and oral availability [14]. Other studies have shown that the content of ginsenosides, the active ingredient of ginseng, varies depending on factors such as variety, origin, and growth years, which makes the quality of ginseng difficult to control. In addition, the dosage of ginseng is also difficult to precisely control, and the excessive consumption of ginseng may cause adverse reactions such as irritation, insomnia, and heart palpitations [15]. In this context, exploring novel methods to enhance the efficacy of active pharmaceutical ingredients in medicinal plants is crucial.

**Figure 1:**
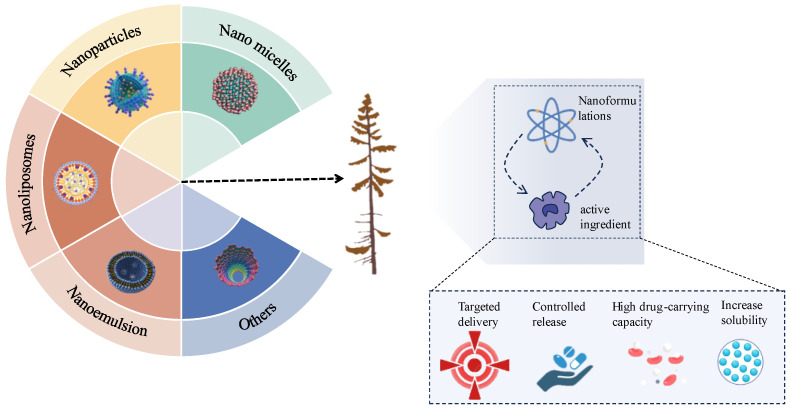
Advantages of improving the efficacy of active pharmaceutical ingredients in medicinal plants through nanoformulations.

In recent years, people’s interest in nanotechnology has shown exponential growth, driving the emergence of multidisciplinary nanomedicine. This field is increasingly being explored to develop new strategies for diagnosing, treating, and preventing diseases, reducing pain, and maintaining and enhancing human health [16,17,18]. Specifically, as an emerging drug delivery system, nanomaterials have shown significant potential in the field of medicinal plants, as shown in Figure 1. Previous studies have shown that the solubility and stability of certain active ingredients in medicinal plants can be significantly improved through nanoformulations. For example, after extracting active ingredients such as paclitaxel, curcumin, luteolin, and quercetin from medicinal plants into nanoformulations, their pharmacological effects have been significantly improved [19,20,21]. Moreover, by combining medicinal plant extracts or active ingredients with nanoformulations, their targeting and triggered release performance can be improved, thereby achieving more effective therapeutic effects [22,23].

The purpose of this review is to systematically summarize the current application status of nanomaterials in enhancing the efficacy of active pharmaceutical ingredients in medicinal plants, analyze the problems and difficulties in the existing research, and explore the future development directions and solutions. By comparing the performance of different nanoformulations, evaluating their pharmacokinetic behavior in vivo, and exploring their interactions with active ingredients in medicinal plants, theoretical support and practical guidance can be provided for further optimizing the efficacy of active pharmaceutical ingredients in medicinal plants.

## 2. Nanoformulations of Medicinal Plant

There are generally the following two forms of development for the nanoformulations of active pharmaceutical ingredients in medicinal plants: nano ultrafine technology and drug nanocarriers [24]. Through nano ultrafine technology such as the recrystallization and pulverization methods (e.g., ball milling and airflow pulverization), traditional Chinese medicines unsuitable for industrial extraction processes, such as mineral or poisonous medicines, and those with easily destructible active ingredients, are finely ground. As the surface area of drug particles expands, a large number of active ingredients are generated, imparting rich physical and chemical properties [25,26]. This method processes and crushes traditional Chinese medicine particles to the nanometer level, representing the initial technology for nano-traditional Chinese medicine. Nanocarrier encapsulation of drugs inherits and develops the ultrafine technology, enabling changes in the drug distribution, regulation of release rate, and enhancement of bioavailability for insoluble drugs [27,28]. Nanodrug carriers achieve passive targeting by enhancing the permeability and retention effects, whereas ligands attached to their surface deliver drugs to specific cells or organs, achieving active targeting [29].

The nano-dosage forms of active pharmaceutical ingredients in medicinal plants primarily include nanoparticles and drug nanocarriers [30]. The nanoparticles of the active pharmaceutical ingredients in medicinal plants encompass nanosuspensions, nano-eutectics, and similar forms, whereas drug nanocarriers exhibit a diverse range of dosage forms, including liposomes, nanoparticles, nanoemulsions, and colloidal polymers [31,32]. In recent years, recent advancements in the research and development of nanocarriers have led to significant progress, including the further modification of carrier surfaces and the incorporation of surface-active agents such as transferrin, folate, low-density lipoprotein, peptides, lectins, and epidermal growth factor. These enhancements notably improve drug targeting, bioavailability, and solubility [33,34,35]. Furthermore, novel nano-based preparations of Chinese medicines, including eutectics, inorganic carriers, phospholipid complexes, and suspension gels, have also been reported [36,37]. The development of nano-dosage forms for active pharmaceutical ingredients in medicinal plants increasingly focuses on enhancing targeting, bioavailability, and solubility.

### 2.1. Nanoliposomes

**Figure 2:**
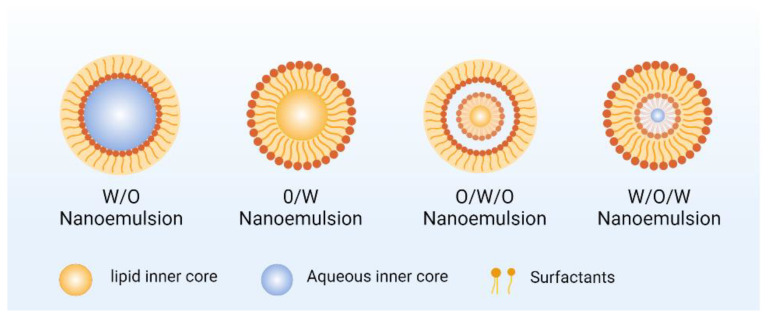
Different types of nanoliposomes.

Nanoliposomes are known as nanoscale bilayer lipid vesicles, typically ranging in size from 20 nm to 200 nm, primarily composed of lipids and phospholipids. They may additionally incorporate other molecules such as carbohydrates, proteins, and sterols [38,39]. As shown in Figure 2, during vesicle formation, nanoliposomes encapsulate both hydrophilic and hydrophobic compounds individually or simultaneously due to their bilayer structure consisting of lipid and aqueous components. Hydrophilic substances are encapsulated within the aqueous regions, including the central core, whereas hydrophobic molecules are integrated into the lipid bilayer membrane or vesicle [40,41]. Compounds encapsulated within nanoliposomes can be released gradually via double-layer diffusion or vesicle degradation triggered by variations in the pH, osmotic pressure, ionic strength, or temperature [39,42,43]. Nanoliposomes that enter the cell through receptor-mediated endocytosis or other endocytic pathways first enter the early endosomal vesicles. The acidic environment in the endosomes can lead to changes in the structure and properties of the nanoliposomes [44]. For example, some pH-sensitive liposomes may release drugs in the acidic environment of the insiders [45]. Subsequently, endosomes may fuse with lysosomes, and nanoliposomes and their encapsulated drugs are transported into lysosomes. In lysosomes, drugs may be degraded by hydrolases, and some nanoliposomes are specially designed to avoid being degraded by lysosomes, so as to achieve the release of drugs in cells [46]. After some nanoliposomes enter cells through caveolin-dependent endocytosis, they first enter the subcellular compartments of the non-lysosomes and eventually diffuse into the cytoplasm; they may further enter organelles such as the Golgi apparatus and endoplasmic reticulum. In these organelles, nanoliposomes and their encapsulated drugs may be involved in some physiological processes within the cell, or they may be secreted outside the cell through the transport mechanism of organelles to achieve cross-cellular transport of drugs or their effects in specific organelles [47,48]. Nanoliposomes loaded with hydrophobic compounds, such as phenolic compounds [49], significantly enhance their solubility and bioavailability, as discussed further in Section 3. Furthermore, the unique bilayer and biofilm structure of nanoliposomes closely resemble natural cell membranes and can mimic their behavior. This capability enables targeted drug delivery into the cells, significantly minimizing damage to the human tissues [50,51]. Nanoliposomes are unquestionably robust and flexible drug delivery systems. Furthermore, their targeting-triggered release and stability properties modify the drug pharmacokinetics and biological distribution, holding substantial promise in tumor therapy [52,53].

### 2.2. Nanoemulsion

Nanoemulsions serve as optimal drug delivery carriers, offering superior encapsulation and delivery capabilities for poorly soluble or lipophilic drugs, thereby shielding them from the impacts of hydrolytic enzymes, gastrointestinal pH, and other environmental conditions [54]. After the nanoemulsion enters the body, it is transported into the bloodstream. Factors such as particle size, surface properties, and binding to plasma proteins affect its stability and circulation time in the blood [55]. Smaller particle sizes and proper surface modifications can reduce the probability of being recognized and cleared by immune system cells such as macrophages, thereby prolonging the time of presence in blood circulation. Nanoemulsions can be enriched in specific tissues or organs by passively targeting or actively targeting mechanisms. Passive targeting takes advantage of the high permeability and retention effect (EPR effect) of the tumor tissues and other sites, making it easier for nanoemulsions to accumulate at these sites. Active targeting involves attaching specific antibodies, ligands, or molecules to the surface of the nanoemulsion so that they can specifically recognize and bind to receptors on target tissues or cells for more precise drug delivery [56,57,58]. These isotropic systems are typically transparent or semi-transparent, with particle sizes ranging from 20 to 500 nm. The drug is evenly distributed within nanodroplets to achieve dispersion, while equilibrium and stability are maintained through the interfacial layer of emulsifiers and co-emulsifiers, ensuring both thermodynamic and kinetic stability [59,60]. Nanoemulsions feature small droplet sizes and possess the capability to solubilize poorly soluble and hydrophobic drugs, thereby enhancing drug solubility and bioavailability [61]. Drug release from nanoemulsions begins with the drug transitioning from the oil phase into a surfactant layer, followed by entry into the aqueous phase. Upon diffusion from the oil, the dissolved drug interfaces with the surrounding water, significantly amplifying its surface area [62].

**Figure 3:**
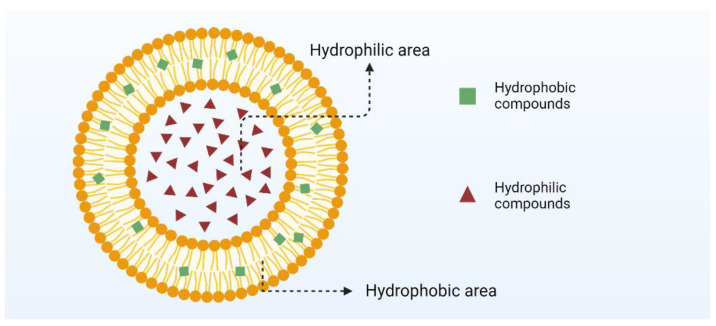
Structure of nanoemulsion.

Nanoemulsions are categorized as oil-in-water, water-in-oil, oil-in-oil, water-in-water bicontinuous emulsions, and nanoemulsions (as depicted in Figure 3). In an oil-in-water emulsion, oil droplets are dispersed within a continuous aqueous phase [62]. In a water-in-oil emulsion, water droplets are dispersed within a continuous oil phase [63]. Water-in-oil nanoemulsions are produced via a two-step high-energy method, involving initial emulsification of oil in the water phase, followed by re-emulsification into the oil phase [60]. These nanoemulsions comprise a multi-chamber system, where water-in-oil emulsions are dispersed as droplets within an external aqueous phase [64]. Water-in-oil nanoemulsions serve as templates for nano gels that encapsulate internal oil droplets within a hydrogel matrix. In the dual-continuous nanoemulsion system, both oil and water droplets are dispersed throughout the system [65].

### 2.3. Nano Micelles

**Figure 4:**
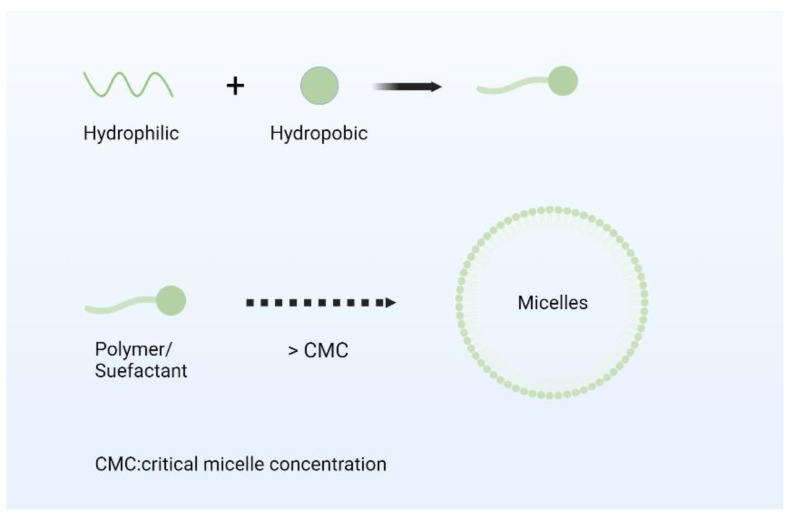
Formation mode of nano micelles.

Nano micelles are typically self-assembled nanoscale colloidal dispersions ranging in size from 10 to 100 nm, characterized by hydrophobic cores and hydrophilic shells, enabling the enhanced solubility and stability for hydrophobic drugs [66,67]. This thermodynamically driven process occurs above a concentration threshold defined by the copolymer, known as the critical micelle concentration [68]. As shown in Figure 4, due to this structural property, micelles are better protected from recognition by the monocyte–macrophage system, prolonging blood circulation. Nano micelles can pass through the gaps between vascular endothelial cells and enter the interstitial space by passive diffusion. Factors such as particle size, surface properties, and hydrophilic/hydrophobic balance affect the efficiency of passive transport. For example, nano micelles with smaller particle sizes and moderate surface hydrophilicity are more likely to enter tissues through the vascular endothelial intercellular space [69,70]. The external hydrophilic corona can be modified with molecules such as transferrin, folate, low-density lipoprotein, peptides, lectins, and epidermal growth factor and its polymer structure can provide additional modification sites to facilitate active targeting [71,72]. Nano micelles encompass both polymer and surfactant micelles, exhibiting varied morphologies such as spherical, tubular, reverse micelle, and bottle brush structures depending on hydrophobicity, hydrophilicity, and solvent conditions. They enhance the cellular uptake of carrier micelles and offer an alternative internalization pathway via endosomes, which is critical in pathology where drug therapy efficacy is affected by drug efflux mechanisms linked to multidrug resistance. These advantages result in significant pharmacokinetic improvements, including prolonged mean residence time in the bloodstream, enhanced bioavailability, precise drug delivery to target tissues, and potentially reduced dosage to mitigate nonspecific organ toxicity.

### 2.4. Nanoparticles

Nanoparticles typically refer to tiny particles ranging from 1 to 100 nm, with their larger surface area and nanoscale size endowing them with unique physicochemical properties [73]. These nanoparticles can consist of single substances such as titanium dioxide, silver nanoparticles, gold nanoparticles, iron oxides, or complexes such as polymer–drug complexes, and nucleic acid–nanoparticle complexes, among others [74,75,76,77]. When nanoparticles enter living organisms, they first come into contact with biological fluids (e.g., blood, tissue fluids, etc.). Various components in biofluids, such as proteins, lipids, sugars, etc., quickly adsorb to the surface of the nanoparticles, forming the so-called “protein corona”. The composition and structure of the protein corona depend on the physicochemical properties of the nanoparticles (e.g., size, shape, surface charge, hydrophilicity, etc.) and the environment of the biofluid (e.g., protein concentration, pH, ionic strength, etc.) [78,79]. For example, gold nanoparticles are smaller in size but have a relatively large specific surface area, which can provide more binding sites for proteins and make it easier to adsorb some low-molecular-weight proteins, such as albumin and transferrin [80]. Polymer nanoparticles with a diameter of several hundred nanometers may bind more to macromolecular proteins such as immunoglobulins [81]. The formation of protein corona will change the surface properties of nanoparticles and affect their subsequent interactions with biological systems, such as biorecognition, immune response, and cellular uptake of nanoparticles [82,83]. Nanoparticles may be recognized by the immune system as a foreign body, triggering an immune response. Immune cells such as macrophages and monocytes can ingest nanoparticles through phagocytosis, which can lead to the removal of nanoparticles before they reach the target site, reducing the efficiency of drug delivery. To avoid this, researchers utilize substances such as folate, protein, polysaccharides, and polyethylene glycol to modify nanoparticle surfaces, thereby designing nanoparticles for targeting specific tissues or cells, improving drug targeting precision while minimizing damage to healthy tissues [84,85]. Compared to other nanoscale delivery systems, nanoparticles prioritize drug protection, precise triggered release, and effective drug loading. They are suitable for water-soluble and sustained-release drugs like peptide drugs that protect them from degradation by gastric acid, ensuring stability and effectiveness in target tissues or cells [86,87].

In addition to the conventional nano delivery systems mentioned above, these include metal–organic frameworks, upconversion nanoparticles, metal nanoelements, and carbon dots. Table 1 classifies these nano-delivery vectors and describes their comparative advantages and limitations.

## 3. Advantages of Nanoformulations in Enhancing the Efficacy of Medicinal Plants

With the continuous advancement of nanotechnology, nanoformulations increasingly play a pivotal role in enhancing the efficacy of active pharmaceutical ingredients in medicinal plants [25]. In addition to the above-mentioned characteristics, nanoformulations exhibit good biocompatibility, helping to weaken the repulsion between drugs and tissues in the body and thereby reducing the side effects on healthy tissues [26]. Based on this, we summarize the advantages of nanoformulations in enhancing the efficacy of active pharmaceutical ingredients in medicinal plants (see Table 2).

### 3.1. Improve Bioavailability and Drug Activity

Flavonoids are crucial polyphenolic antioxidants present in various parts of medicinal plants, including the roots, stems, leaves, and flowers [130,131]. As secondary metabolites, they are synthesized and accumulated throughout the growth process of these plants [132]. The unique structure of flavonoids not only endows them with defensive, protective, and communicative functions in plants but also imparts medicinal value [133]. However, the hydrophobic nature of most flavonoids results in low bioavailability, which hinders their clinical applications. Recently, combining flavonoids with nano-delivery systems has been anticipated to offer novel strategies to address this challenge.

Baicalin, a flavonoid component derived from the traditional Chinese medicine Huangcen, is primarily extracted from the dried roots, stems, and leaves of this plant. It exhibits a range of pharmacological effects commonly associated with traditional Chinese medicine, including antibacterial, antiviral, antioxidant, anti-inflammatory, and anti-tumor properties [134,135]. Recent studies suggest that baicalin has potential applications in both chemotherapy and immunotherapy, and it is anticipated to emerge as a novel anti-tumor agent with dual chemotherapy and immunotherapy effects. However, as a chemotherapeutic agent, baicalin suffers from poor solubility and necessitates high doses to achieve significant tumoricidal effects. At elevated doses, baicalin is prone to cumulative toxicity and other adverse side effects [136,137,138]. Additionally, baicalin is a small-molecule flavonoid glycoside characterized by low hydrophilicity and lipophilicity. It is insoluble in water at room temperature, resulting in poor water solubility and consequently low bioavailability [139]. The low water solubility and inadequate absorption of baicalin constrain its clinical applicability.

Nanoparticle-based drug delivery systems are effective methods for enhancing drug efficacy. Various nanoparticle formulations of baicalin have been explored, including nanocrystals, nanoemulsions, nanoliposomes, nano micelles, and nanophospholipid complexes, to improve their bioavailability and solubility. Mi et al. [140] developed a baicalin nanoparticle drug delivery system using zeolite imidazole framework-8 (ZIF-8) as a carrier. Notably, 4T1 breast cancer cells were inoculated into female BALB/c mice for in vivo experiments. By degrading the outer layer of polyethylene glycol–folate–folate in an acidic environment with reduced pH in the cancer cells, the ligand bonds of ZIF-8 were also gradually broken in the acidic environment, thereby slowly releasing the drug and inhibiting the growth of the cancer cells. Wei et al. [141] were the first to develop a baicalin-loaded nanoliposome system for targeted lung delivery. In vitro evaluations revealed that the baicalin-loaded nanoliposomes exhibited high encapsulation efficiency and exceptional stability. Nude mice carrying orthotopic human lung cancer cells were injected intravenously with the same volume of blank nanoliposomes, baicalin solution (100 mg baicalin/kg body weight), and baicalin nanoliposomes (100 mg baicalin/kg body weight); in vivo evaluations demonstrated that the baicalin-loaded nanoliposomes significantly increased the drug concentration in the lungs following a single intravenous administration. Notably, the baicalin-loaded nanoemulsion appeared to be more effective than the other nanoformulations. Xu et al. [142] developed a water-in-oil nanoemulsion loaded with baicalin. By examining the degree of lymphatic uptake in rats, it was found that compared with baicalin suspension, the bioavailability of baicalin in the nanoemulsion increased, and its detectable concentration in vivo was sustained for at least 12 h. This enhancement may be attributed to the increased permeability induced by surfactants and co-surfactants, sustained release of baicalin, and augmented uptake through lymphatic transport [142,143]. From this, it can be seen that baicalin nanoformulations are more effective than traditional formulations, exhibiting better absorption and higher bioavailability.

Naringin, a type of citrus flavonoid, exhibits diverse biological activities such as antioxidant, anti-diabetic, anticancer, anti-inflammatory, antidepressant, anti-obesity, anti-hypertensive, and cardiovascular disease prevention. Despite these health benefits, Naringin’s therapeutic potential remains constrained by its low water solubility and poor permeability [111,112,118]. Being hydrophobic, naringin exhibits low solubility in aqueous buffers, approximately 475 mg/L [113]. The oral bioavailability of naringin in both animals and humans is less than 10% [144], possibly due to reduced gastrointestinal absorption involving passive diffusion and active transport mechanisms [145].

Recent studies on encapsulating naringin with biopolymers have garnered significant attention among researchers. Biopolymers are preferred over other nanocarriers for delivering hydrophobic flavonoids like naringin due to their biocompatibility, biodegradability, and slower release rates compared to low molecular weight surfactants [146,147]. One study showed that naringenin was loaded into the hydrophobic core of bovine beta-casein micelles through hydrophobic interactions. In this process, the naringenin molecule bound tightly to the hydrophobic region of the bovine beta-casein micelle and was stably encapsulated inside the micelle. Naringenin-loaded bovine beta-casein micelles exhibited lower critical micelle concentrations and higher aggregation numbers, resulting in a significant increase in their concentrations in aqueous solutions [117]. Smruthi et al. [94] loaded naringin with P/P-Nar NPs; the hydrophobic nature of naringenin allowed it to interact with the hydrophobic region of zein and be encapsulated inside the zein nanoparticles or nanofibers. At the same time, the hydrophobic core of casein micelles can also accommodate naringenin molecules and the loading of naringenin can be realized through hydrophobic interactions, demonstrating significant alterations in naringin’s physicochemical properties and bioavailability under simulated gastrointestinal conditions. Compared to free naringin, P/P-Nar NPs increased their bioavailability by 4.7 times in the rat model. In addition to the characteristics of general biopolymers, the mucosal adhesion of chitosan can make the nanopolymers stay on the mucosal surface for a long time, improve the absorption efficiency of drugs, encapsulation efficiency of naringenin exceeds 90%, and cause significant anti-diabetic reactions after oral administration [110]. Thus, biopolymers represent a promising approach as effective nanocarriers for delivering naringin.

Quercetin is one of the most extensively studied flavonoids, found abundantly in fruits and vegetables. Quercetin exhibits notable biological activities, including antioxidant, anticancer, and anti-inflammatory effects. Leveraging its antioxidant activity, quercetin can activate and bolster endogenous defense mechanisms against free radicals [105,148]. Despite its significant biological potential, quercetin’s clinical application is limited by its extremely low solubility, poor stability, and low bioavailability, with solubility levels of only 5.5 μg/mL in gastric fluids and 28.9 μg/mL in intestinal fluids. The oral bioavailability of quercetin in rats is less than 17%, while in humans, it is approximately 1%, which limits its application in pharmaceutical formulations [108,149].

Quercetin, being a lipid-soluble nutrient, can be encapsulated within the hydrophobic core of these lipid particles [55,107]. At the same time, it can also be adsorbed at the interface between the oil phase and the aqueous phase, and the adsorption layer formed by the surfactant and co-surfactant at the interface can provide a stable environment for quercetin to prevent its aggregation and precipitation [106]. Incorporating quercetin into a particle transport system can enhance its solubility, stability, bioavailability, and overall biological activity. Research indicates that nanoemulsion can enhance the bioavailability of fat-soluble substances by facilitating rapid and complete digestion in the small intestine [88]. Du et al. [104] developed four delivery systems—crude emulsion, nanoemulsion, high internal phase emulsion, and emulsion gel—to transport quercetin and identify the most effective system in a simulated gastrointestinal tract. The results show that the average particle size of the nanoemulsion is relatively small, and the droplet distribution is relatively uniform, which gives the nanoemulsion the characteristics of difficult aggregation after heat treatment. On the other hand, the absolute ζ potential of the nanoemulsion is relatively high, indicating that there is a high electrostatic repulsion between the droplets, which helps to maintain its stability by reducing the possibility of droplet coalescence caused by droplet contact, and the nanoemulsion has good thermal stability and storage stability. The results demonstrate that nanoemulsion exhibits superior thermal and storage stability. Compared to other delivery systems, nanoemulsion is the optimal choice for quercetin delivery due to its higher bioavailability. Additionally, Sathishkumar et al. [114] developed a zinc oxide–quercetin nanocomposite as an advanced nanomedicine delivery system. The quercetin loading capacity on ZnO nanoparticles was 210 μg/mg, with an IC 50 value of 0.01 μg/mL on MCF-7 cancer cells (compared to 0.07 μg/mL for free quercetin), demonstrating potent anti-cancer effects. Hemati et al. used niosome (a bilayer nonionic surfactant-based vesicle that is a novel drug delivery system) as a vehicle to deliver quercetin. Hydrophobic quercetin was encapsulated within the lipid layer, and cytotoxicity experiments showed that the IC50s of quercetin, loaded by this delivery system, were significantly lower than those of the free form [150]. Embedding quercetin within a nanoscale particle transport system proved to be an effective approach.

Similarly, many studies have shown that nanomaterials enhance the bioavailability and drug activity of other flavonoids. Yi et al. [109] modified puerarin with unsaturated olefins using acryloyl chloride and obtained an amphiphilic polymer called poly puerarin via free radical polymerization, which was used to prepare drug delivery systems. They also prepared poly puerarin nanoparticles using the nanoprecipitation method, addressing the low solubility and low oral utilization of puerarin. Demirturk et al. [115] analyzed daidzein in rat plasma, and the relative bioavailability of soybean flavonoid nanoformulations prepared with nanoemulsion and nanosuspension reached 265.6% and 262.3%, respectively. The research results of Hamadou et al. [93] indicated that nanoliposomes have good encapsulation efficiency for rutin, and coating these nanoliposomes loaded with rutin with multiple layers of pectin and chitosan can enhance the protection of bioactive compounds and nanoliposomes from various environmental stresses, thereby expanding their potential applications. Self-assembling nano gas carriers based on non-invasive effervescence effectively enhances the solubility of low water-soluble luteolin [151]. It should be pointed out that although various preclinical mechanism studies have been conducted on the nanoformulations of flavonoids, there is a lack of carefully designed randomized clinical trials on the therapeutic activity and safety of such nanoformulations, emphasizing the need for more clinical research.

### 3.2. Trigger Release

These stimulus signals are primarily categorized into two types, namely internal stimuli and external stimuli [152]. Internal stimuli include factors such as pH value, temperature, hypoxia, enzymatic processes, and glutathione concentration. Significant environmental changes between the normal and tumor tissues affect the pH values, redox conditions, and malignant biological molecular properties. Tumor tissues exhibit higher temperatures, acidic pH values, elevated concentrations of glutathione, and overexpression of specific enzymes compared to normal tissues [153,154]. Due to these intrinsic gradients, internal stimulus signals act as ideal activators for triggering drug release systems and enhancing their precision in targeting tumor tissues. However, external stimuli, including light, magnetic fields, electric fields, and ultrasound-based drug delivery systems (DDS), provide triggered drug release and reduce individual variability compared to internal stimulus parameters [23,155]. This paper primarily discusses the effects of pH and light on the control of drug delivery systems.

pH. In internal stimulation, pH is one of the most commonly used triggers for drug release in triggered release systems, as different pathologies exhibit varying pH values during their progression [156]. Moreover, when nanomaterials enter cells, they encounter variations in cellular pH, such as in tumor and inflammatory tissues (pH~6.8), endosomes (pH~5.5–6), and lysosomes (pH~4.5–5.0). Therefore, pH-responsive triggered release systems offer a safe and effective means of regulating drug release in specific regions of the body. Recently, successful examples of pH-responsive triggered release systems utilizing nanomaterials have been developed. After receiving a specific pH stimulation, polymer nanocarriers can achieve a triggered release not only through matrix degradation or diffusion but also through their own interactions with the drug, such as hydrogen bonding, hydrophobic interactions, or electrostatic interactions, to affect the release rate of the drug. The molecular arrangement of a drug in a polymer determines its release behavior [157]. Aniruhan et al. [158] treated curcumin-loaded drug delivery systems with simulated gastric acid pH (1.2), acidic tumor pH (5.0), and simulated intestinal fluid pH (7.4), and performed drug release studies on the curcumin-loaded polymer carriers loaded with curcumin at different pH values. Curcumin adsorbed on the surface of the polymer carrier became protonated due to the presence of imines, carboxyl groups, and hydroxyl groups in the material. This protonation induced electron repulsion within the material, leading to swelling and the enhanced release of curcumin. Within 48 h, the drug release reached 91.0% at the acidic tumor pH of 5.5, which is significantly higher compared to the release percentages at pH 1.2 and 7.4. Gong et al. [159] prepared mesoporous zinc oxide nanocarriers using polyoxyethylene–polyoxypropylene ether block copolymer (Poloxamer 188) and sucrose as dual templates for loading tea polyphenols. Under weak acidic conditions, the release rate and proportion of tea polyphenols in the nanocomposites were significantly improved. This use of hydrogels as polymer nanocarriers is characterized by a three-dimensional network structure filled with water, where the tea polyphenols can be released by diffusion in the pores and channels of the hydrogel, and the hydrogel can swell in response to pH. At pH 5.5, the release rate and proportion of the nanocomposites reached the highest level, with a maximum equilibrium release rate of 90%. In addition to this, studies on the release of ZnO–quercetin nanocomposites showed that the hard ligand (-OH group) of quercetin remained in its ionized form at pH = 7.4. Thus, it can act as an active ligand for chelation formation. At pH 5.5, the ZnO–quercetin complex is unstable because the OH group of ZnO–quercetin exists in a combined form and also partially dissolves the ZnO nanoparticles, this suggests that quercetin is released more rapidly under the acidic conditions (typical cancer pH 5.5) compared to the physiological conditions (pH 7.4) [114]. Thus, these nanoformulations exhibit pH sensitivity and can be precisely triggered for release at pH levels adapted by cancer cells, making them more suitable for cancer treatment.

Light. Light is regarded as a distinctive exogenous triggering factor, operating within a broad, effective, and relatively safe energy range—primarily infrared, ultraviolet, and visible light—and can induce molecular changes to activate and release drugs in a fully cell-compatible manner [160,161]. It can also be highly focused to induce local reactions, thereby enabling precise spatiotemporal control over therapeutic release [162]. Given these intrinsic characteristics, light has consistently been one of the most favored external triggering factors in therapeutic applications. Upconversion nanoparticles have unique upconversion luminescence properties that absorb low-energy near-infrared light and convert it into high-energy visible or ultraviolet light. This property allows for the upconversion nanoparticles to be excited in deep tissues, as near-infrared light penetrates more deeply into biological tissues, reducing damage to the surrounding healthy tissues [95]. Hu et al. [163] developed a near-infrared photoresponsive nanomedicine delivery system utilizing carvacrol. The system comprises upconversion nanoparticles that can convert 808 nm near-infrared light to blue light, mesoporous silica (serving as the carrier channel), and hydrophobic carvacrol (exhibiting blue light-responsive properties). Under 808 nm near-infrared irradiation, the upconversion nanoparticles display specific antibacterial (including against anaerobic bacteria), anti-inflammatory, and immunomodulatory properties due to the synergistic effect of carvacrol and upconverted blue light. Wen et al. [164] synthesized a novel pH and near-infrared responsive nanocomposite with ZIF-8 as the carrier. This material encapsulates the upconversion nanoparticles and titanium dioxide nanoparticles, with energy upconverted through the photosensitive interaction between the chemotherapy agents, curcumin and TiO_2_. In vivo experiments in rats have shown that ZIF-8 decomposes in the acidic microenvironment of tumors, facilitating the conversion of irradiated near-infrared 808 nm light into ultraviolet-visible light by TiO_2_ NPs, thereby generating a substantial amount of reactive oxygen species and effectively inhibiting tumors. In addition, photosensitizer-mediated lipid oxidation is an effective drug-triggered release mechanism, photosensitizers can absorb molecules of specific wavelengths of light, use their own reactive oxygen species to trigger lipid oxidation reactions, and destroy the structure of lipid membranes, so as to achieve precise drug release [127]. Meerovich et al. [165] developed a paclitaxel-loaded solid lipid nanoparticle system using low-intensity (23 mW/cm^2^) near-infrared (approximately 730 nm) illumination as the light source. Within 4 h, in a cellular model of A549 in vitro, the paclitaxel release from these solid lipid nanoparticles was less than 10%, increasing eightfold after zero-point illumination. This validates the feasibility of combining herbal monomers with photoresponsive in situ nano drug delivery systems, offering a more effective and reliable strategy for potential applications in deep tissue diseases.

Multi-Response Triggered Release System. In addition to the single-response triggered release system mentioned above, researchers have also developed a series of triggered release systems capable of responding to complex environmental stimuli. These multi-response systems offer numerous potential applications in the further development of intelligent triggered release systems. Li et al. [166] developed a microgel loaded with gambogic acid that exhibits triple environmental responsiveness. This system not only accelerates drug release to target tumor cells at an acidic pH, thereby reducing agent presence and high temperatures, but also demonstrates fewer side effects and greater safety compared to free gambogic acid. Hafezi et al. [167] constructed a novel biocompatible nanocarrier utilizing magnetic nano-sensitizers as luminescent agents (ZnFe_2_O_4_@mZnO-N-GQDs and ZnFe_2_O_4_@mZnO-GQDs). This nanocarrier was used for curcumin-triggered release, with pH and ultrasound-triggered intelligent drug release, and incorporated N-GQDs (nitrogen-doped graphene quantum dots) to enhance biocompatibility and hydrophilicity. The controlled and sustained release of curcumin from nanocarriers, triggered by pH and ultrasound, may help reduce the side effects on normal tissues while increasing tumor selectivity. In summary, this reliable and direct strategy for establishing a multifunctional drug delivery platform opens new avenues for developing innovative anti-tumor therapy delivery systems suitable for clinical application.

### 3.3. Targeted Action

The targeting of nanomedicine can enable drugs to reach target organs and cells more effectively than non-targeted delivery methods, thereby significantly improving therapeutic efficacy [154]. The concept of the “Property flavors attributive channel” in traditional Chinese medicine refers to the selectivity of drugs towards specific organs and meridians, which can enhance their affinity for targeted areas of the body [168]. The integration of nanotechnology can leverage this affinity, allowing the design of targeted drug delivery systems that maintain a specific drug concentration at the target site and introduce messenger drugs to enhance targeting synergistically [22,169]. Targeted drug delivery systems are generally classified into passive targeting, active targeting, and responsive targeting.

Passive targeting. Due to vascular leakage and impaired lymphatic clearance, small molecule compounds are efficiently extravasive and retained in the tumor stroma (enhanced permeability and retention (EPR) effect). Passive targeting refers to the passive accumulation of small molecules through the EPR effect, thereby achieving a therapeutic effect in tumor tissues [170]. Nano drugs (20–200 nm) can accumulate in tumors because they can pass through the gaps between tumor vascular endothelial cells (200–2000 nm) and are retained due to defects in the lymphatic circulation within the tumor tissue [171,172]. For instance, egg yolk lecithin liposomes with a size of 100 nm circulate in mice for up to 12.8 h. A longer circulation time results in greater accumulation at the tumor site [173]. Resveratrol, as a non-flavonoid polyphenolic compound found in a large number of medicinal plants, has anti-inflammatory and antioxidant properties. Over the past three decades, it has also been found to exhibit anti-cancer properties in some cancers, such as liver, breast, and ovarian cancer. However, resveratrol is less water-soluble and has limited dissolution in the aqueous environment of the gastrointestinal tract. It is mainly absorbed in the gastrointestinal tract by passive diffusion, but due to its molecular structure characteristics, it is relatively difficult to transport across cell membranes. There are also a variety of metabolic enzymes in the gastrointestinal tract and liver, such as the cytochrome P450 enzyme system. Resveratrol is easily metabolized into other metabolites by these metabolic enzymes during gastrointestinal absorption and hepatic first-pass metabolism. These metabolites may have different bioactive and pharmacokinetic properties, resulting in a reduction in the original active ingredient of resveratrol and a decrease in bioavailability [174,175]. Yao et al. [125] used polylactic acid–glycolic acid copolymer nanoparticles as carriers for resveratrol loading, and in vitro experiments showed that this specific nano delivery system has the characteristics of good stability, long vascular circulation time, etc., and increased toxicity of resveratrol to cancer cells, which is especially suitable for passive targeted therapy of tumors. Patra et al. [102] prepared a resveratrol-loaded gold nanoparticle. The large surface area of spherical gold nanoparticles enabled a relatively high loading of resveratrol molecules, which also increased the intermolecular binding between surface molecules and preloaded drugs. In mice carrying breast cancer cells, resveratrol-loaded gold nanoparticles, were retained longer in the colon tumors than in normal colon tissues, suggesting that resveratrol-loaded gold nanoparticles had improved targeting of colon tumors. In addition, Vijayakumar et al. used D-α-tocopheryl polyethylene glycol 1000 succinate-coated solid lipid nanoparticles loaded with resveratrol and found that this nanocarrier could passively target gliomas after intravenous administration to Charles Foster rats [176]. Among them, the brain distribution of resveratrol loaded with solid lipid nanoparticles was 9.23 times higher than that of resveratrol alone, and there was no accumulation in the surrounding tissues, suggesting that the nanoloaded system could reduce the damaging effect on other tissues and sites. It is worth noting that although the EPR effect can endow nanomaterials with the ability to accumulate in the tumor area, the degree of angiogenesis and pore size in the tumor is closely related to the type and state of the tumor, which significantly affects the targeting efficiency. For example, breast cancer typically has higher levels of angiogenesis and larger vascular pores, allowing nanomaterials to penetrate and accumulate more easily. However, the vascular network of liver cancer may be more complex and irregular, resulting in poor penetration of nanomaterials [177]. In addition, in some sclerosing tumors, such as pancreatic cancer, the high density of the vascular matrix limits the penetration of nanomaterials [178]. Therefore, although passive targeting based on the EPR effect can selectively accumulate at the lesion site and enhance the local therapeutic effect of the drug, its universality is limited [179].

Active targeting. To enhance both the interaction between the drug carriers and tumor cells and the internalization of these carriers by tumor cells, it is essential to incorporate active targeting functions into the drug carriers. Active targeting refers to the combination of targeted portions of the nanocarrier surface, such as aptamers, ligands, and antibodies, with specific recognition of tumor cells. By specifically recognizing overexpressed receptors on the surface of tumor cells, it stimulates receptor-mediated endocytosis to internalize nanocarriers, resulting in better therapeutic outcomes [22]. Active targeting involves modifying the surface of nanomedicines with ligands, thereby significantly improving their specificity for targeted cells and reducing off-target drug release compared to passive targeting. Ligands such as folate, transferrin, biotin, and hyaluronic acid are overexpressed in certain cells. Nanocarriers that bind to specific receptors for these ligands can effectively enhance the targeting capability of traditional Chinese medicine. For example, in normal tissue cells, folic acid enters the cell through the transmembrane transport, while in cancer cells, the folate receptors on the cell surface recognize folic acid and form a folate complex, at which point endocytic vesicles are formed to enter cancer cells through endocytosis, which is the main way that folic acid molecules recognize and enter cancer cells [180,181]. Based on the fact that folic acid enters normal cells and tumor cells in different ways, it can be modified to the surface of the carrier as a targeting molecule, so that the drug delivery system can selectively enter tumor cells and release loaded drugs in the cytoplasm, play the role of anticancer agents in biomedicine, and realize targeted therapy for tumors [120,182]. Chen et al. [120] developed a folic acid-modified nano-herbal micelle for the delivery of paclitaxel and intravenous injection to nude mice transplanted with A549 cells. This actively targeted nano micelles demonstrating excellent tumor-targeting properties with minimal toxicity. Essa et al. [116] prepared polymeric nanoparticles from polylactic acid–glycolic acid copolymers for quercetin loading and used folic acid as a ligand modification to investigate whether it could provide selective toxicity and enhance the uptake in model LnCap prostate cancer cells. By comparing free quercetin with the corresponding non-targeted system, it was found that the folic acid-targeted nanosystem increased the uptake and toxicity of cancer cells. Li et al. [14] prepared 4-aminophenyl β-D-galactopyranoside/mulberry leaf polysaccharide lysozyme/luteolin nanoparticles via amide reaction, self-assembly, and electrostatic interaction. In an in vitro HepG2 cell study, this nanoparticle targets the liver for luteolin delivery and enhances its efficacy in liver tissue by specifically recognizing sialic acid protein receptors, thereby increasing the concentration of luteolin. Curcumin-loaded liposomes modified with galactose could recognize specific stem cells, thereby enhancing curcumin’s anti-tumor efficacy [183]. Additionally, particles such as cells, vesicles, and viruses in biomimetic drug delivery systems offer novel approaches for enhancing drug targeting. Extracellular vesicle-like natural nanovesicles from Camellia contain various bioactive polyphenolic compounds, including tea catechin gallate esters and epicatechin gallate. In vivo experiments have demonstrated that extracellular vesicle-like natural nanovesicles exhibit strong targeting ability due to their accumulation at the tumor and metastasis sites [184], thereby providing new possibilities for developing novel nanocarriers.

Responsive targeting. Responsive targeting is a method that uses a specific stimulus-response mechanism to enable drug carriers to change in specific physiological or pathological environments in vivo so as to achieve precisely targeted delivery to lesion sites. It is different from the two targeted reactions mentioned above. Responsive drug carriers can precisely control the rate and amount of drug release based on the intensity and duration of the stimulus, enabling on-demand release. This is difficult to achieve with passive targeting and active targeting, which can better meet the needs of treatment, improve the efficacy of drugs, and reduce the side effects of drugs [185]. For example, Wang et al. [186] developed a method for loading HM (a β-carboline extracted from the traditional Chinese herb Peganum harmala) into the Au@MSNs@PEG@Asp6 (NPS) nanocomposite material. This material exhibits low cytotoxicity to normal tissues, good biocompatibility, and can precisely target lung adenocarcinoma cells through its responsiveness to reactive oxygen species, which facilitates the release of HM and effectively reduces the migration and invasion of these cancer cells. Moreover, responsive targeting can be tailored to individual physiological conditions or disease characteristics to achieve personalized treatment. For example, in the tumor microenvironment, glutathione levels are significantly higher compared to normal tissues [187]. Ma et al. [90] designed a glutathione-sensitive nano micelle that binds to the polyphenolic compound curcumin for the treatment of esophageal cancer. Glutathione in the tumor microenvironment stimulates the release of curcumin, thereby enhancing its targeted delivery efficiency to the tumor site. Gene-specific drug delivery is also a component of personalized therapy. Multidrug resistance protein-1 (MDR-1), also known as P-glycoprotein, is a member of the ATP-binding cassette transporters that can reduce the intracellular accumulation of chemotherapy drugs and induce chemotherapy resistance [188]. Yang et al. [189] designed an N-succinyl chitosan–lipoic acid micelle for the co-delivery of paclitaxel and MDR1-siRNA. As siRNA downregulates MDR1, the intracellular concentration of paclitaxel increases, allowing for continuous targeting and accumulation in diseased tissues, which is crucial for inhibiting cancer progression. This drug release strategy can achieve more precise therapeutic effects, offering new possibilities for treating complex diseases and enhancing patients’ quality of life.

## 4. Limitations and Potential Toxicity of Nanoformulations of Medicinal Plants

Although numerous nano-functional formulations of active pharmaceutical ingredients in medicinal plants have been developed, most research remains confined to the laboratory, with few advancing to clinical application. To effectively address the challenges associated with nanoformulations, the following factors must be considered:

(1) The particle size and distribution of nanoformulations have an important impact on their performance and biological effects. In large-scale production, it is difficult to ensure that every batch of nanoparticles has a uniform particle size and a narrow particle size distribution. Small fluctuations in the production process, such as changes in temperature, stirring speed, reaction time, etc., can lead to uneven particle size [190]. For example, in the preparation of nanoparticles by emulsion polymerization, agitation that is too fast may result in smaller particle sizes but, at the same time, increase the width of the particle size distribution. Many methods for the preparation of nanoformulations can be successfully implemented in the laboratory, but various problems are encountered when scaling up to industrial-scale production. For example, some nano-preparation methods based on microfluidic technology can accurately control the flow and reaction conditions of fluids in the laboratory to prepare high-quality nano-preparations, but in large-scale production, microfluidic equipment is expensive and complex to operate, making it difficult to achieve industrial application [191,192].
(2) There is still a lot of uncertainty about the long-term toxicity and potential risks of nanomaterials. In vivo, the nanoformulations may interact with biomolecules, cells, and tissues, producing some unexpected biological effects. For example, nanoparticles may accumulate in the body, causing damage to the organs such as the liver and kidneys; or trigger an immune response, leading to adverse reactions such as allergies and inflammation [193,194]. However, due to the relatively short development and application time of nanoformulations, the long-term toxicity and potential risks of nanoformulations have not been sufficiently studied, which poses a great challenge to the safety assessment of regulatory authorities. In addition, the biocompatibility of nanoformulations is also a key issue. Some nanomaterials may interact with the immune system, blood system, etc., affecting the normal physiological functions of organisms. For example, certain nanoparticles may activate the complement system, leading to the formation of immune complexes and inflammatory responses; or bind to proteins in the blood, altering the rheological properties of the blood and increasing the risk of thrombosis [195,196].
(3) The pharmacokinetic behavior of nanoformulations in vivo is very different from that of conventional drugs. Factors such as the size, shape, and surface properties of nanoparticles affect their distribution, metabolism, and excretion in the body. For example, nanoparticles with smaller particle sizes may be more likely to enter the interstitial space through the walls of blood vessels, while nanoparticles with specific ligands on the surface can enable targeted delivery to specific tissues or cells. Traditional medicines are metabolized primarily by hepatic metabolic enzymes. Nanoformulations may be taken up by immune cells such as macrophages, where different metabolic processes occur within the cell. For example, some lipid nanoformulations may be degraded by lysosomes within the cells [46]. However, these unique in vivo behaviors make traditional pharmacokinetic research methods no longer applicable, and new research methods and models need to be developed to accurately assess the pharmacokinetic properties of nanoformulations.
(4) At present, the international regulatory standards for nanoformulations are not uniform, and there are differences in regulatory requirements in different countries and regions. This makes enterprises face great confusion when carrying out the research and development and declaration of nanoformulations and increases the difficulty and cost of listing products in different regions. The established (and in some cases, unwanted regulations) set by drug regulatory authorities are another challenge on the road to new nano dosage forms. Regulations from regulatory agencies such as the NMPA, EMA, and FDA are different from one another, and these regulations are always subject to change on a regular basis. As a result, such regulations can significantly impact the entire process of a clinical trial.

Overall, numerous challenges persist regarding the full experimental and clinical translation of nanoformulations of active pharmaceutical ingredients in medicinal plants. Consequently, a comprehensive understanding of key issues, including quality control and safety assessment in nanomedicine, as well as the close integration of basic chemistry, pharmacology, and materials science, is essential. Clear strategies must be developed to address the challenges associated with medicinal plant nanomaterials effectively.

## 5. Conclusions

This review provides a comprehensive and in-depth analysis of the promising field of enhancing the efficacy of active pharmaceutical ingredients in medicinal plants through nanotechnology-based formulations. It highlights the significant potential of these nanoformulations in addressing medical needs and advancing the modernization of traditional Chinese medicine. The study of nanoformulations reveals that they offer innovative and effective methods for improving the efficacy of active pharmaceutical ingredients in medicinal plants. Nanotechnology notably enhances the solubility, stability, and bioavailability of medicinal plant components, improves drug targeting, and thus increases therapeutic efficacy. Detailed research into the interaction between nanomaterials and active pharmaceutical ingredients in medicinal plants has uncovered complex mechanisms involving nanocarriers and the active ingredients of these plants. This synergistic effect not only optimizes drug release patterns but may also activate the latent therapeutic potential of medicinal plant components, offering both the theoretical foundations and practical guidance for developing more efficient nanoformulations of active pharmaceutical ingredients in medicinal plants. Nonetheless, the limitations and potential toxicity associated with nanoformulations of active pharmaceutical ingredients in medicinal plants must be acknowledged. In practical applications, the preparation process of nanoformulations requires further optimization to achieve standardization in large-scale production and quality control. Additionally, potential toxicity issues necessitate long-term, comprehensive research to evaluate their impact on human health. Despite the current challenges, the considerable potential of nanomaterials to enhance the efficacy of active pharmaceutical ingredients in medicinal plants cannot be overlooked. Future research should focus on addressing existing problems, exploring innovative strategies for integrating nanotechnology with traditional Chinese medicine and fostering interdisciplinary collaboration to advance this field. With ongoing technological advancements and rigorous research, nanoformulations of active pharmaceutical ingredients in medicinal plants are expected to play a more significant role in traditional Chinese medicine and contribute substantially to human health. In summary, research into enhancing the efficacy of active pharmaceutical ingredients in medicinal plants through nanoformulations is a promising and evolving field that holds the potential to open new applications for medicinal plants and leads to significant breakthroughs in the pharmaceutical industry.

## Figures and Tables

**Table 1:** Comparative advantages and limitations of various nanocarriers.

Type	Comparative Advantage	Limitations	References
Nanoemulsion	Strong drug-loading capacity. The particle size is small and uniform. High bioavailability.	Potential toxicity of surfactants; the storage conditions are demanding.	[59,60,62,88]
Nano micelles	High loading capacity, good stability in blood, prolonged circulation time, low number of side effects, and protects internal drugs from degradation.	Stability still needs to be improved; complex behavior in vivo	[89,90]
Nanoliposomes	Passive targeting of drugs, highly efficient cargo delivery, reducing cargo toxicity.	The storage and transportation conditions are demanding; stability issues.	[38,91,92,93,94]
Upconversion nanoparticles	Unique optical properties, low toxicity and good biocompatibility, and easy surface functionalization.	The optical conversion efficiency needs to be improved. The drug load is relatively low.	[95]
Metal-organic framework	High specific surface area and porosity, biodegradability, structure and performance can be adjusted.	Synthesis and preparation are complex. There are limited studies on drug loading and release kinetics.	[96,97]
Nanometallic elemental	Low cytotoxicity, controlled size and surface, easy synthesis, high cell permeability, ability to bind many molecules on their surface, high drug-loading capacity.	Clearance problems in the body; difficult to achieve complex drug delivery patterns.	[98,99]
Carbon dots	Very high elastic modulus and mechanical strength, high electrical and thermal conductivity, prolonged circulating time, cell membrane permeability, high aspect ratio allowing for high drug loading.	Limited drug loading. Preparation is complex and costly.	[100]

**Table 2:** Improving the bioavailability and drug activity of active pharmaceutical ingredients in medicinal plants through nanoformulations.

Nanoformulations	Active Ingredient	Impacts	Reference
Nanoemulsion	Curcumin	Compared with curcumin dispersed in conventional hydrogel systems, the developed curcumin nanolatex exhibits thixotropic rheological behavior with a significant increase in skin permeability.	[12,49,101,102]
Nanoemulsion	Brucine	Brucine-loaded nanolatex exhibits superior anti-inflammatory and antinociceptive activity in reducing hind paw swelling and inhibiting acetic acid-induced abdominal writhing compared to brucine-loaded gels or brucine-loaded latex.	[54,55,60,61,63,103]
Nanoemulsion	Quercetin	In four delivery systems, the highest bioavailability of quercetin was observed in nanoemulsions.	[104,105,106,107,108]
Nanoparticles	Puerarin	Compared with free puerarin, poly-puerarin nanoparticles have the best anti-tumor effect.	[72,76,77,109]
Nanoparticles	Naringenin	In experimental rat models, naringenin-loaded nanoparticles were more effective than free naringenin in improving Streptozotocin-induced diabetogenic effects.	[94,110,111,112,113]
Nanoparticles	Quercetin	The amount of quercetin loaded on the ZnO nanoparticles reached 210 μg/mg, and the half maximal inhibitory concentration value of ZnO-quercetin nanocomposites for Michigan Cancer Foundation-7 breast cancer cells was 0.01 (0.07 μg/mL for free quercetin).	[51,105,114,115,116]
Nano micelles	Naringenin	Compared with pure bovine beta-casein micelles, naringenin-containing bovine beta-casein micelles had a lower critical micelle concentration and a larger aggregation number, which greatly increased the concentration of naringenin in aqueous solution.	[111,112,117,118]
Nano micelles	Podophyllotoxin	Compared with the other two cytotoxic agent-loaded micelles, podophyllotoxin-loaded micelles had the highest activity.	[89,119,120]
Nano micelles	Thymoquinone	Thymoquinone polymer micelles exhibit better wound-healing effects than natural thymoquinone and silver sulfadiazine.	[90,120,121]
Nanoliposomes	*Sargassum boveanum*	After encapsulating the extract with nanoliposomes, *Sargassum boveanum* retained a high proportion of phenolic compounds for antioxidant properties.	[92,93,122]
Nanoliposomes	Capsaicin	Compared with capsaicin, nanoliposomes encapsulating capsaicin have improved pharmacokinetic properties, enhanced anticancer activity, and selectivity.	[38,91,123]
Nanoliposomes	Lutin	Better protection of the vitality of rutin bioactive compounds.	[52,93,123]
Nanocrystals	Resveratrol	Compared to nonnanocrystalline forms, resveratrol nanocrystals exhibit better anti-tumor effects than resveratrol itself.	[102,124,125]
Nanocrystals	Isoliquiritigenin	Compared with the free form of isoliquiritigenin, it has higher solubility and lower toxicity to cells.	[126,127,128]
Nanocrystals	Breviscapine	Compared with brevisanthin microparticle formulations, brevisanthin nanocrystals can provide relatively stable drug concentrations in plasma for a long time.	[124,129]
Nanocrystals	Ginkgolide B	Ginkgolide B nanocrystals show higher drug plasma levels and neuronal drug distribution compared to free ginkgolide B.	[127,128]

## References

[1] Wang K.L., Chen Q., Shao Y.Y., Yin S.S., Liu C.Y., Liu Y.M., Wang R., Wang T., Qiu Y.L., Yu H.Y. (2021). Anticancer activities of TCM and their active components against tumor metastasis. Biomed. Pharmacother..

[2] WHO (2019). Selection and Use of Essential Medicines: Report of the WHO Expert Committee on Selection and Use of Essential Medicines, 2019 (including the 21st WHO Model List of Essential Medicines and the 7th WHO Model List of Essential Medicines for Children). Selection and Use of Essential Medicines: Report of the WHO Expert Committee on Selection and Use of Essential Medicines.

[3] Tripathy V., Basak B.B., Varghese T.S., Saha A. (2015). Residues and contaminants in medicinal herbs—A review. Phytochem. Lett..

[4] Theodoridis S., Drakou E.G., Hickler T., Thines M., Nogues-Bravo D. (2023). Evaluating natural medicinal resources and their exposure to global change. Lancet Planet. Health.

[5] Vaou N., Stavropoulou E., Voidarou C., Tsigalou C., Bezirtzoglou E. (2021). Towards Advances in Medicinal Plant Antimicrobial Activity: A Review Study on Challenges and Future Perspectives. Microorganisms.

[6] Fan P.H., Wu L.W., Wang Q., Wang Y., Luo H.M., Song J.Y., Yang M.H., Yao H., Chen S.L. (2023). Physiological and molecular mechanisms of medicinal plants in response to cadmium stress: Current status and future perspective. J. Hazard. Mater..

[7] Lv G.S., Li Z.H., Zhao Z.Y., Liu H.L., Li L., Li M.H. (2024). The factors affecting the development of medicinal plants from a value chain perspective. Planta.

[8] Shan Z.J., Ye J.F., Hao D.C., Xiao P.G., Chen Z.D., Lu A.M. (2022). Distribution patterns and industry planning of commonly used traditional Chinese medicinal plants in China. Plant Divers..

[9] Li Z.G., Wang Y.X., Xu M.W., Liu H.Y., Li L., Xu D.L. (2023). Molecular mechanism overview of metabolite biosynthesis in medicinal plants. Plant Physiol. Biochem..

[10] Noor F., ul Qamar M.T., Ashfaq U.A., Albutti A., Alwashmi A.S.S., Aljasir M.A. (2022). Network Pharmacology Approach for Medicinal Plants: Review and Assessment. Pharmaceuticals.

[11] Shedoeva A., Leavesley D., Upton Z., Fan C. (2019). Wound Healing and the Use of Medicinal Plants. Evid.-Based Complement. Altern. Med..

[12] Tomeh M.A., Hadianamrei R., Zhao X.B. (2019). A Review of Curcumin and Its Derivatives as Anticancer Agents. Int. J. Mol. Sci..

[13] Chen S.L., Yu H., Luo H.M., Wu Q., Li C.F., Steinmetz A. (2016). Conservation and sustainable use of medicinal plants: Problems, progress, and prospects. Chin. Med..

[14] Li R.L., Zhou J.N., Zhang X.Y., Wang Y.J., Wang J., Zhang M., He C.W., Zhuang P.W., Chen H.X. (2023). Construction of the Gal-NH2/mulberry leaf polysaccharides-lysozyme/ luteolin nanoparticles and the amelioration effects on lipid accumulation. Int. J. Biol. Macromol..

[15] Hu Y.B., Li Y.M., Cao Y., Shen Y.Z., Zou X.J., Liu J.X., Zhao J. (2024). Advancements in enzymatic biotransformation and bioactivities of rare ginsenosides: A review. J. Biotechnol..

[16] Donthi M.R., Munnangi S.R., Krishna K.V., Saha R.N., Singhvi G., Dubey S.K. (2023). Nanoemulgel: A Novel Nano Carrier as a Tool for Topical Drug Delivery. Pharmaceutics.

[17] Liu X.L., Dong X., Yang S.C., Lai X., Liu H.J., Gao Y.H., Feng H.Y., Zhu M.H., Yuan Y.H., Lu Q. (2021). Biomimetic Liposomal Nanoplatinum for Targeted Cancer Chemophototherapy. Adv. Sci..

[18] Kong B., Liu R., Guo J.H., Lu L., Zhou Q., Zhao Y.J. (2023). Tailoring micro/nano-fibers for biomedical applications. Bioact. Mater..

[19] Reda F.M., El-Saadony M.T., Elnesr S.S., Alagawany M., Tufarelli V. (2020). Effect of Dietary Supplementation of Biological Curcumin Nanoparticles on Growth and Carcass Traits, Antioxidant Status, Immunity and Caecal Microbiota of Japanese Quails. Animals.

[20] Qu N., Wang C.Y., Meng Y.M., Gao Y.H. (2023). Superior Anticancer Potential of Nano-Paclitaxel Combined Bevacizumab Treatment in Ovarian Cancer. Curr. Pharm. Biotechnol..

[21] Bi F.Y., Qin Y., Chen D., Kan J., Liu J. (2021). Development of active packaging films based on chitosan and nano-encapsulated luteolin. Int. J. Biol. Macromol..

[22] Shi P.Z., Cheng Z.R., Zhao K.C., Chen Y.H., Zhang A.R., Gan W.K., Zhang Y.K. (2023). Active targeting schemes for nano-drug delivery systems in osteosarcoma therapeutics. J. Nanobiotechnol..

[23] Adepu S., Ramakrishna S. (2021). Controlled Drug Delivery Systems: Current Status and Future Directions. Molecules.

[24] Bonifácio B.V., da Silva P.B., Ramos M.A.D., Negri K.M.S., Bauab T.M., Chorilli M. (2014). Nanotechnology-based drug delivery systems and herbal medicines: A review. Int. J. Nanomed..

[25] Kumar M., Keshwania P., Chopra S., Mahmood S., Bhatia A. (2023). Therapeutic Potential of Nanocarrier-Mediated Delivery of Phytoconstituents for Wound Healing: Their Current Status and Future Perspective. Aaps Pharmscitech.

[26] Bernela M., Seth M., Kaur N., Sharma S., Pati P.K. (2023). Harnessing the potential of nanobiotechnology in medicinal plants. Ind. Crops Prod..

[27] Churilov G., Ivanycheva J., Kiryshin V. Influence of Nano-Crystal Metals on Texture and Biological Properties of Water Soluble Polysaccharides of Medicinal Plants. Proceedings of the 3rd International Youth Conference on Interdisciplinary Problems of Nanotechnology, Biomedicine and Nanotoxicology (Nanobiotech).

[28] Barman A., Kotal A., Das M. (2023). Synthesis of metal based nano particles from Moringa Olifera and its biomedical applications: A review. Inorg. Chem. Commun..

[29] Liu Y.L., Yang Y., Zhang Q.R., Lu D.H., Li S.Y., Li J.F., Yang G.C., Shan Y.P. (2021). Dynamics of delivering aptamer targeted nano-drugs into cells. J. Mater. Chem. B.

[30] Rajput D., Singh M., Sahu P., Jain D., Kashaw S.K., Patil U.K. (2023). Advances in Nanogel as Drug Delivery System for Cancer Therapeutics: An Overview. Mini-Rev. Med. Chem..

[31] Pretorius D., Serpooshan V., Zhang J.Y. (2021). Nano-Medicine in the Cardiovascular System. Front. Pharmacol..

[32] Zou J.H., Li M., Liu Z.W., Luo W., Han S.Q., Xiao F., Tao W., Wu Q.B., Xie T., Kong N. (2024). Unleashing the potential: Integrating nano-delivery systems with traditional Chinese medicine. Nanoscale.

[33] Lopus M. (2023). Nano-ayurvedic medicine and its potential in cancer treatment. J. Integr. Med. JIM.

[34] Dai Y.X., Wang Y.H., Yang Z. (2021). Anti-inflammatory effects of traditional chinese medicine ingredients based on nano-Strychnos liposomes for external skin application. Ferroelectrics.

[35] Shang Q., Liu W.D., Leslie F., Yang J.P., Guo M.M., Sun M.J., Zhang G.J., Zhang Q., Wang F.H. (2024). Nano-formulated delivery of active ingredients from traditional Chinese herbal medicines for cancer immunotherapy. Acta Pharm. Sin. B.

[36] Kang C.L., Wang J.W., Li R.T., Gong J.N., Wang K.R., Wang Y.X., Wang Z.H., He R.Z., Li F.Y. (2023). Smart Targeted Delivery Systems for Enhancing Antitumor Therapy of Active Ingredients in Traditional Chinese Medicine. Molecules.

[37] Zheng Y.H., Wang Y., Xia M.Y., Gao Y., Zhang L., Song Y.N., Zhang C. (2022). The combination of nanotechnology and traditional Chinese medicine (TCM) inspires the modernization of TCM: Review on nanotechnology in TCM-based drug delivery systems. Drug Deliv. Transl. Res..

[38] Faghihi H., Mozafari M.R., Bumrungpert A., Parsaei H., Taheri S.V., Mardani P., Dehkharghani F.M., Pudza M.Y., Alavi M. (2023). Prospects and challenges of synergistic effect of fluorescent carbon dots, liposomes and nanoliposomes for theragnostic applications. Photodiagn. Photodyn. Ther..

[39] Akhavan S., Assadpour E., Katouzian I., Jafari S.M. (2018). Lipid nano scale cargos for the protection and delivery of food bioactive ingredients and nutraceuticals. Trends Food Sci. Technol..

[40] Farjadian F., Ghasemi A., Gohari O., Roointan A., Karimi M., Hamblin M.R. (2019). Nanopharmaceuticals and nanomedicines currently on the market: Challenges and opportunities. Nanomedicine.

[41] Azarashkan Z., Farahani S., Abedinia A., Akbarmivehie M., Motamedzadegan A., Heidarbeigi J., Hayaloglu A.A. (2022). Co-encapsulation of broccoli sprout extract nanoliposomes into basil seed gum: Effects on in vitro antioxidant, antibacterial and anti-*Listeria* activities in ricotta cheese. Int. J. Food Microbiol..

[42] Ozpolat B., Sood A.K., Lopez-Berestein G. (2014). Liposomal siRNA nanocarriers for cancer therapy. Adv. Drug Deliv. Rev..

[43] Ashrafizadeh M., Delfi M., Zarrabi A., Bigham A., Sharifi E., Rabiee N., Paiva-Santos A.C., Kumar A.P., Tan S.C., Hushmandi K. (2022). Stimuli-responsive liposomal nanoformulations in cancer therapy: Pre-clinical & clinical approaches. J. Control. Release.

[44] Shirane M. (2020). Lipid Transfer-Dependent Endosome Maturation Mediated by Protrudin and PDZD8 in Neurons. Front. Cell Dev. Biol..

[45] Vanic Z., Barnert S., Süss R., Schubert R. (2012). Fusogenic activity of PEGylated pH-sensitive liposomes. J. Liposome Res..

[46] Hamer I., Van Beersel G., Arnould T., Jadot M. (2012). Lipids and Lysosomes. Curr. Drug Metab..

[47] Albright J.M., Sydor M.J., Shannahan J., Ferreira C.R., Holian A. (2023). Imipramine Treatment Alters Sphingomyelin, Cholesterol, and Glycerophospholipid Metabolism in Isolated Macrophage Lysosomes. Biomolecules.

[48] Jaishy B., Abel E.D. (2016). Lipids, lysosomes, and autophagy. J. Lipid Res..

[49] Kumari A., Raina N., Wahi A., Goh K.W., Sharma P., Nagpal R., Jain A., Ming L.C., Gupta M. (2022). Wound-Healing Effects of Curcumin and Its Nanoformulations: A Comprehensive Review. Pharmaceutics.

[50] Agrawal M., Ajazuddin, Tripathi D.K., Saraf S., Saraf S., Antimisiaris S.G., Mourtas S., Hammarlund-Udenaes M., Alexander A. (2017). Recent advancements in liposomes targeting strategies to cross blood-brain barrier (BBB) for the treatment of Alzheimer’s disease. J. Control. Release.

[51] Homayoonfal M., Aminianfar A., Asemi Z., Yousefi B. (2024). Application of Nanoparticles for Efficient Delivery of Quercetin in Cancer Cells. Curr. Med. Chem..

[52] Li Q.B., Lv L.A., Liang W.Q., Chen Z.B., Deng Q., Sun L.J., Wang Y.L., Liu Y. (2024). Screening, characterization and mechanism of a potential stabiliser for nisin nanoliposomes with high encapsulation efficiency. Food Chem..

[53] Huang M.G., Cong L.X., Ying R.F., Ahmad M., Hao G., Hayat K., Salamatullah A.M. (2024). Polysaccharide-coated quercetin-loaded nanoliposomes mitigate bitterness: A comparison of carrageenan, pectin, and trehalose. Int. J. Biol. Macromol..

[54] Rai V.K., Mishra N., Yadav K.S., Yadav N.P. (2018). Nanoemulsion as pharmaceutical carrier for dermal and transdermal drug delivery: Formulation development, stability issues, basic considerations and applications. J. Control. Release.

[55] Zhang H.C., Deng L.A., Yang J., Yang G.W., Fan H.T., Yin Y.Q., Luo S., Li S.S., Liu L.Y., Yang M. (2023). Preparation and evaluation of a nanoemulsion containing cordycepin and its protective effect on skin. J. Dispers. Sci. Technol..

[56] Deshpande D., Kethireddy S., Gattacceca F., Amiji M. (2014). Comparative pharmacokinetics and tissue distribution analysis of systemically administered 17-β-estradiol and its metabolites in vivo delivered using a cationic nanoemulsion or a peptide-modified nanoemulsion system for targeting atherosclerosis. J. Control. Release.

[57] Choudhary A., Jain P., Mohapatra S., Mustafa G., Ansari M.J., Aldawsari M.F., Alalaiwe A.S., Mirza M.A., Iqbal Z. (2022). A Novel Approach of Targeting Linezolid Nanoemulsion for the Management of Lymph Node Tuberculosis. ACS Omega.

[58] Yang S.L., Lin H.S., Zhang L., Ho P.C.L. (2024). Formulating 10-hydroxycamptothecin into nanoemulsion with functional excipient tributyrin: An innovative strategy for targeted hepatic cancer chemotherapy. Int. J. Pharm..

[59] Gazolu-Rusanova D., Lesov I., Tcholakova S., Denkov N., Ahtchi B. (2020). Food grade nanoemulsions preparation by rotor-stator homogenization. Food Hydrocoll..

[60] Sneha K., Kumar A. (2022). Nanoemulsions: Techniques for the preparation and the recent advances in their food applications. Innov. Food Sci. Emerg. Technol..

[61] Jaiswal M., Dudhe R., Sharma P.K. (2015). Nanoemulsion: An advanced mode of drug delivery system. 3 Biotech.

[62] Shehabeldine A.M., Doghish A.S., El-Dakroury W.A., Hassanin M.M.H., Al-Askar A.A., AbdElgawad H., Hashem A.H. (2023). Antimicrobial, Antibiofilm, and Anticancer Activities of *Syzygium aromaticum* Essential Oil Nanoemulsion. Molecules.

[63] Choi S.J., McClements D.J. (2020). Nanoemulsions as delivery systems for lipophilic nutraceuticals: Strategies for improving their formulation, stability, functionality and bioavailability. Food Sci. Biotechnol..

[64] Chu Y.F., Gao C.C., Liu X.Y., Zhang N., Xu T., Feng X., Yang Y.L., Shen X.C., Tang X.Z. (2020). Improvement of storage quality of strawberries by pullulan coatings incorporated with cinnamon essential oil nanoemulsion. LWT-Food Sci. Technol..

[65] Liu Y.C., Zhao L.T., Chen H.Y., Ye Z.M., Guo L., Zhou Z.Q. (2024). Nobiletin enhances the antifungal activity of eugenol nanoemulsion against Penicillium italicum in both in vitro and in vivo settings. Int. J. Food Microbiol..

[66] Cagel M., Tesan F.C., Bernabeu E., Salgueiro M.J., Zubillaga M.B., Moretton M.A., Chiappetta D.A. (2017). Polymeric mixed micelles as nanomedicines: Achievements and perspectives. Eur. J. Pharm. Biopharm..

[67] Avramovic N., Mandic B., Savic-Radojevic A., Simic T. (2020). Polymeric Nanocarriers of Drug Delivery Systems in Cancer Therapy. Pharmaceutics.

[68] Perumal S., Atchudan R., Lee W. (2022). A Review of Polymeric Micelles and Their Applications. Polymers.

[69] Chen D.Q., Wang G.H., Song W.G., Zhang Q. (2015). Novel CD44 receptor targeting multifunctional “nano-eggs” based on double pH-sensitive nanoparticles for co-delivery of curcumin and paclitaxel to cancer cells and cancer stem cells. J. Nanopart. Res..

[70] Delorme V., Lichon L., Mahindad H., Hunger S., Laroui N., Daurat M., Godefroy A., Coudane J., Gary-Bobo M., Van den Berghe H. (2020). Reverse poly(ε-caprolactone)-*g*-dextran graft copolymers. Nano-carriers for intracellular uptake of anticancer drugs. Carbohydr. Polym..

[71] Cabral H., Kataoka K. (2014). Progress of drug-loaded polymeric micelles into clinical studies. J. Control. Release.

[72] Elmowafy M., Shalaby K., Elkomy M.H., Alsaidan O.A., Gomaa H.A.M., Abdelgawad M.A., Mostafa E.M. (2023). Polymeric Nanoparticles for Delivery of Natural Bioactive Agents: Recent Advances and Challenges. Polymers.

[73] Muhamad N., Plengsuriyakarn T., Na-Bangchang K. (2018). Application of active targeting nanoparticle delivery system for chemotherapeutic drugs and traditional/herbal medicines in cancer therapy: A systematic review. Int. J. Nanomed..

[74] Joseph T.M., Mahapatra D.K., Esmaeili A., Piszczyk L., Hasanin M.S., Kattali M., Haponiuk J., Thomas S. (2023). Nanoparticles: Taking a Unique Position in Medicine. Nanomaterials.

[75] Yazdanian M., Rostamzadeh P., Rahbar M., Alam M., Abbasi K., Tahmasebi E., Tebyaniyan H., Ranjbar R., Seifalian A., Yazdanian A. (2022). The Potential Application of Green-Synthesized Metal Nanoparticles in Dentistry: A Comprehensive Review. Bioinorg. Chem. Appl..

[76] Khan Y., Sadia H., Shah S.Z.A., Khan M.N., Shah A.A., Ullah N., Ullah M.F., Bibi H., Bafakeeh O.T., Ben Khedher N. (2022). Classification, Synthetic, and Characterization Approaches to Nanoparticles, and Their Applications in Various Fields of Nanotechnology: A Review. Catalysts.

[77] Selmani A., Kovacevic D., Bohinc K. (2022). Nanoparticles: From synthesis to applications and beyond. Adv. Colloid Interface Sci..

[78] Zhang Z.B., Ren J.J., Dai W.B., Zhang H., Wang X.Q., He B., Zhang Q. (2023). Fast and Dynamic Mapping of the Protein Corona on Nanoparticle Surfaces by Photocatalytic Proximity Labeling. Adv. Mater..

[79] Rezaei G., Daghighi S.M., Haririan I., Yousefi I., Raoufi M., Rezaee F., Dinarvand R. (2019). Protein corona variation in nanoparticles revisited: A dynamic grouping strategy. Colloids Surf. B Biointerfaces.

[80] García-Alvarez R., Hadjidemetriou M., Sánchez-Iglesias A., Liz-Marzán L.M., Kostarelos K. (2018). In vivo formation of protein corona on gold nanoparticles. The effect of their size and shape. Nanoscale.

[81] Natte K., Friedrich J.F., Wohlrab S., Lutzki J., von Klitzing R., Österle W., Orts-Gil G. (2013). Impact of polymer shell on the formation and time evolution of nanoparticle-protein corona. Colloids Surf. B Biointerfaces.

[82] Singh U., Saifi Z., Kumar M., Reimers A., Krishnananda S.D., Adelung R., Baum M. (2021). Role of structural specificity of ZnO particles in preserving functionality of proteins in their corona. Sci. Rep..

[83] Sun H.A., Wang Y.Y., Zhang L.M. (2023). Tuning the Microstructure of Protein Corona by Nanoparticle Hydrophobicity: A Dissipative Particle Dynamics Study. Chem. Lett..

[84] Dai L., Sun C.X., Li R.R., Mao L.K., Liu F.G., Gao Y.X. (2017). Structural characterization, formation mechanism and stability of curcumin in zein-lecithin composite nanoparticles fabricated by antisolvent co-precipitation. Food Chem..

[85] Luther D.C., Huang R., Jeon T., Zhang X.Z., Lee Y.W., Nagaraj H., Rotello V.M. (2020). Delivery of drugs, proteins, and nucleic acids using inorganic nanoparticles. Adv. Drug Deliv. Rev..

[86] Yetisgin A.A., Cetinel S., Zuvin M., Kosar A., Kutlu O. (2020). Therapeutic Nanoparticles and Their Targeted Delivery Applications. Molecules.

[87] Anselmo A.C., Mitragotri S. (2015). A Review of Clinical Translation of Inorganic Nanoparticles. AAPS J..

[88] McClements D.J. (2021). Advances in edible nanoemulsions: Digestion, bioavailability, and potential toxicity. Prog. Lipid Res..

[89] Jo M.J., Jin I.S., Park C.W., Hwang B.Y., Chung Y.B., Kim J.S., Shin D.H. (2020). Revolutionizing technologies of nanomicelles for combinatorial anticancer drug delivery. Arch. Pharm. Res..

[90] Ma Z.M., Gao X.Z., Raza F., Zafar H., Huang G.H., Yang Y.Y., Shi F., Wang D.Q., He X. (2022). Design of GSH-Responsive Curcumin Nanomicelles for Oesophageal Cancer Therapy. Pharmaceutics.

[91] Zarrabi A., Abadi M.A.A., Khorasani S., Mohammadabadi M.R., Jamshidi A., Torkaman S., Taghavi E., Mozafari M.R., Rasti B. (2020). Nanoliposomes and Tocosomes as Multifunctional Nanocarriers for the Encapsulation of Nutraceutical and Dietary Molecules. Molecules.

[92] Mozafari M.R., Weissig V. (2010). Nanoliposomes: Preparation and Analysis. Liposomes: Methods and Protocols, Volume 1: Pharmaceutical Nanocarriers.

[93] Hamadou A.H., Zhang J.Y., Chao C., Xu B. (2022). Stability of rutin using pectin-chitosan dual coating nanoliposomes. LWT-Food Sci. Technol..

[94] Smruthi M.R., Nallamuthu I., Anand T. (2022). A comparative study of optimized naringenin nanoformulations using nano-carriers (PLA/PVA and zein/pectin) for improvement of bioavailability. Food Chem..

[95] Ovais M., Mukherjee S., Pramanik A., Das D., Mukherjee A., Raza A., Chen C.Y. (2020). Designing Stimuli-Responsive Upconversion Nanoparticles that Exploit the Tumor Microenvironment. Adv. Mater..

[96] Chen X.L., Hong L.H., Wu Y., Gu Y.C., Luo J.G., Kong L.Y. (2024). A dual recognition-based strategy employing Ni-modified metal-organic framework for in situ screening of SIRT1 inhibitors from Chinese herbs. Talanta.

[97] Bazzazan S., Moeinabadi-Bidgoli K., Lalami Z.A., Bazzazan S., Mehrarya M., Yeganeh F.E., Hejabi F., Akbarzadeh I., Noorbazargan H., Jahanbakhshi M. (2023). Engineered UIO-66 metal-organic framework for delivery of curcumin against breast cancer cells: An in vitro evaluation. J. Drug Deliv. Sci. Technol..

[98] Xie Y.L., Liu H.K., Xie X.L., Li Y., Peng F., Zhao Y., Xu H. (2023). Active ingredients biosynthesis and genic response of traditional Chinese medicine *Ligusticum sinense* cv. Chuanxiong under different cadmium level. Ind. Crops Prod..

[99] Sharma R., Bhardwaj R., Gautam V., Kohli S.K., Kaur P., Bali R.S., Saini P., Thukral A.K., Arora S., Vig A.P., Egamberdieva D., Ahmad P. (2018). Microbial Siderophores in Metal Detoxification and Therapeutics: Recent Prospective and Applications. Plant Microbiome: Stress Response.

[100] Lai H.H., Ming P.T., Liu Y.X., Wang S.M., Zhou Q., Zhai H.Y. (2023). MWCNTs and ZnO-based Ce-MOF nanocomposites as enhanced sensing platform for electrochemical detection of carbendazim in Chinese traditional herbs samples. Microchim. Acta.

[101] Algahtani M.S., Ahmad M.Z., Nourein I.H., Albarqi H.A., Alyami H.S., Alyami M.H., Alqahtani A.A., Alasiri A., Algahtani T.S., Mohammed A.A. (2021). Preparation and Characterization of Curcumin Nanoemulgel Utilizing Ultrasonication Technique for Wound Healing: In Vitro, Ex Vivo, and In Vivo Evaluation. Gels.

[102] Patra S., Pradhan B., Nayak R., Behera C., Rout L., Jena M., Efferth T., Bhutia S.K. (2021). Chemotherapeutic efficacy of curcumin and resveratrol against cancer: Chemoprevention, chemoprotection, drug synergism and clinical pharmacokinetics. Semin. Cancer Biol..

[103] Abdallah M.H., Abu Lila A.S., Unissa R., Elsewedy H.S., Elghamry H.A., Soliman M.S. (2021). Preparation, characterization and evaluation of anti-inflammatory and anti-nociceptive effects of brucine-loaded nanoemulgel. Colloids Surf. B Biointerfaces.

[104] Du X.Q., Hu M., Liu G.N., Qi B.K., Zhou S.J., Lu K.Y., Xie F.Y., Zhu X.Q., Li Y. (2022). Development and evaluation of delivery systems for quercetin: A comparative study between coarse emulsion, nano-emulsion, high internal phase emulsion, and emulsion gel. J. Food Eng..

[105] Kandemir K., Tomas M., McClements D.J., Capanoglu E. (2022). Recent advances on the improvement of quercetin bioavailability. Trends Food Sci. Technol..

[106] Dario M.F., Oliveira C.A., Cordeiro L.R.G., Rosado C., Mariz I.D.A., Maçôas E., Santos M., da Piedade M.E.M., Baby A.R., Velasco M.V.R. (2016). Stability and safety of quercetin-loaded cationic nanoemulsion: In vitro and in vivo assessments. Colloids Surf. A Physicochem. Eng. Asp..

[107] Marques B.M., Machado P.A., Santos A.P., Carrett-Dias M., Araújo S.G., Alves B.D., de Oliveira B.S., da Silva F.M.R., Dora L.C., Cañedo D.A. (2021). Anti-MDR Effects of Quercetin and its Nanoemulsion in Multidrug-Resistant Human Leukemia Cells. Anti-Cancer Agents Med. Chem..

[108] Zou H.Y., Ye H.Q., Kamaraj R., Zhang T.H., Zhang J., Pavek P. (2021). A review on pharmacological activities and synergistic effect of quercetin with small molecule agents. Phytomedicine.

[109] Yi T.Q., Huang J., Chen X.W., Xiong H.Y., Kang Y., Wu J. (2019). Synthesis, characterization, and formulation of poly-puerarin as a biodegradable and biosafe drug delivery platform for anti-cancer therapy. Biomater. Sci..

[110] Maity S., Mukhopadhyay P., Kundu P.P., Chakraborti A.S. (2017). Alginate coated chitosan core-shell nanoparticles for efficient oral delivery of naringenin in diabetic animals-An in vitro and in vivo approach. Carbohydr. Polym..

[111] Motallebi M., Bhia M., Rajani H.F., Bhia I., Tabarraei H., Mohammadkhani N., Pereira-Silva M., Kasaii M.S., Nouri-Majd S., Mueller A.L. (2022). Naringenin: A potential flavonoid phytochemical for cancer therapy. Life Sci..

[112] Moghaddam R.H., Samimi Z., Moradi S.Z., Little P.J., Xu S.W., Farzaei M.H. (2020). Naringenin and naringin in cardiovascular disease prevention: A preclinical review. Eur. J. Pharmacol..

[113] Stabrauskiene J., Kopustinskiene D.M., Lazauskas R., Bernatoniene J. (2022). Naringin and Naringenin: Their Mechanisms of Action and the Potential Anticancer Activities. Biomedicines.

[114] Sathishkumar P., Li Z.F., Govindan R., Jayakumar R., Wang C.Y., Gu F.L. (2021). Zinc oxide-quercetin nanocomposite as a smart nano-drug delivery system: Molecular-level interaction studies. Appl. Surf. Sci..

[115] Demirturk E., Kaplan A.B.U., Cetin M., Akillioglu K., Kutlu M.D., Kose S., Aksu F. (2022). Assessment of Pharmacokinetic Parameters of Daidzein-Containing Nanosuspension and Nanoemulsion Formulations After Oral Administration to Rats. Eur. J. Drug Metab. Pharmacokinet..

[116] Essa D., Kondiah P.P.D., Kumar P., Choonara Y.E. (2023). Design of Chitosan-Coated, Quercetin-Loaded PLGA Nanoparticles for Enhanced PSMA-Specific Activity on LnCap Prostate Cancer Cells. Biomedicines.

[117] Su M., Dai Q.X., Chen C., Zeng Y., Chu C.C., Liu G. (2020). Nano-Medicine for Thrombosis: A Precise Diagnosis and Treatment Strategy. Nano-Micro Lett..

[118] Wang Q.Y., Ou Y.J., Hu G.M., Wen C., Yue S.S., Chen C., Xu L., Xie J.W., Dai H., Xiao H. (2020). Naringenin attenuates non-alcoholic fatty liver disease by down-regulating the NLRP3/NF-κB pathway in mice. Br. J. Pharmacol..

[119] Kalinova R.G., Dimitrov I.V., Ivanova D.I., Ilieva Y.E., Tashev A.N., Zaharieva M.M., Angelov G., Najdenski H.M. (2024). Polycarbonate-Based Copolymer Micelles as Biodegradable Carriers of Anticancer Podophyllotoxin or Juniper Extracts. J. Funct. Biomater..

[120] Chen C., Du S.Y., Zhong W., Liu K.G., Qu L.H., Chu F.Y., Yang J.J., Han X. (2022). Accurate delivery of pristimerin and paclitaxel by folic acid-linked nano-micelles for enhancing chemosensitivity in cancer therapy. Nano Converg..

[121] Negi P., Sharma G., Verma C., Garg P., Rathore C., Kulshrestha S., Lal U.R., Gupta B., Pathania D. (2020). Novel thymoquinone loaded chitosan-lecithin micelles for effective wound healing: Development, characterization, and preclinical evaluation. Carbohydr. Polym..

[122] Feng Y., Zarei V., Mousavipour N. (2023). Provision and assessment properties of nanoliposomes containing macroalgae extracts of *Sargassum boveanume* and *Padina pavonica*. LWT Food Sci. Technol..

[123] Al-Samydai A., Alshaer W., Al-Dujaili E.A.S., Azzam H., Aburjai T. (2021). Preparation, Characterization, and Anticancer Effects of Capsaicin-Loaded Nanoliposomes. Nutrients.

[124] Ancic D., Orsolic N., Odeh D., Tomasevic M., Pepic I., Ramic S. (2022). Resveratrol and its nanocrystals: A promising approach for cancer therapy?. Toxicol. Appl. Pharmacol..

[125] Yao F., Lin L.Z., Shi W., Li C.S., Liang Z.J., Huang C.L. (2022). Inhibitory Effect of Poly(lactic-co-glycolic acid) Nanoparticles Loaded with Resveratrol and Phosphatase and Tensin Homolog Deleted on Chromosome Ten (PTEN) siRNA on Lung Cancer Cells. Sci. Adv. Mater..

[126] Qiao F.X., Zhao Y., Zhang W.N., Yang J.H. (2020). Isoliquiritigenin Nanosuspension Enhances Cytostatic Effects in A549 Lung Cancer Cells. Planta Med..

[127] Wang H.J., Shrestha R., Zhang Y. (2014). Encapsulation of Photosensitizers and Upconversion Nanocrystals in Lipid Micelles for Photodynamic Therapy. Part. Part. Syst. Charact..

[128] Liu Y., Liu W., Xiong S., Luo J.S., Li Y., Zhao Y.Y., Wang Q., Zhang Z.X., Chen X.J., Chen T.K. (2020). Highly stabilized nanocrystals delivering Ginkgolide B in protecting against the Parkinson’s disease. Int. J. Pharm..

[129] Zhang T., Li X., Xu J., Shao J., Ding M., Shi S. (2022). Preparation, Characterization, and Evaluation of Breviscapine Nanosuspension and Its Freeze-Dried Powder. Pharmaceutics.

[130] Yu P., He X.M., Baer M., Beirinckx S., Tian T., Moya Y.A.T., Zhang X.C., Deichmann M., Frey F.P., Bresgen V. (2021). Plant flavones enrich rhizosphere Oxalobacteraceae to improve maize performance under nitrogen deprivation. Nat. Plants.

[131] Yan D.W., Tajima H., Cline L.C., Fong R.Y., Ottaviani J., Shapiro H.Y., Blumwald E. (2022). Genetic modification of flavone biosynthesis in rice enhances biofilm formation of soil diazotrophic bacteria and biological nitrogen fixation. Plant Biotechnol. J..

[132] Zhao C.N., Liu X.J., Gong Q., Cao J.P., Shen W.X., Yin X.R., Grierson D., Zhang B., Xu C.J., Li X. (2021). Three AP2/ERF family members modulate flavonoid synthesis by regulating type IV chalcone isomerase in citrus. Plant Biotechnol. J..

[133] Bangar S.P., Kajla P., Chaudhary V., Sharma N., Ozogul F. (2023). Luteolin: A flavone with myriads of bioactivities and food applications. Food Biosci..

[134] Huang T., Liu Y.A., Zhang C.L. (2019). Pharmacokinetics and Bioavailability Enhancement of Baicalin: A Review. Eur. J. Drug Metab. Pharmacokinet..

[135] Kong N., Chen X.Y., Feng J., Duan T., Liu S.P., Sun X.N., Chen P., Pan T., Yan L.L., Jin T. (2021). Baicalin induces ferroptosis in bladder cancer cells by downregulating FTH1. Acta Pharm. Sin. B.

[136] Fan Z.Y., Cai L.L., Wang S.N., Wang J., Chen B.H. (2021). Baicalin Prevents Myocardial Ischemia/Reperfusion Injury Through Inhibiting ACSL4 Mediated Ferroptosis. Front. Pharmacol..

[137] Hu Q.C., Zhang W.W., Wu Z., Tian X., Xiang J.B., Li L.X., Li Z.H., Peng X., Wei S.Z., Ma X. (2021). Baicalin and the liver-gut system: Pharmacological bases explaining its therapeutic effects. Pharmacol. Res..

[138] Jin X., Liu M.Y., Zhang D.F., Zhong X., Du K., Qian P., Yao W.F., Gao H., Wei M.J. (2019). Baicalin mitigates cognitive impairment and protects neurons from microglia-mediated neuroinflammation via suppressing NLRP3 inflammasomes and TLR4/NF-κB signaling pathway. CNS Neurosci. Ther..

[139] Singh S., Meena A., Luqman S. (2021). Baicalin mediated regulation of key signaling pathways in cancer. Pharmacol. Res..

[140] Mi X., Hu M.G., Dong M.R., Yang Z.H., Zhan X., Chang X.Y., Lu J., Chen X. (2021). Folic Acid Decorated Zeolitic Imidazolate Framework (ZIF-8) Loaded with Baicalin as a Nano-Drug Delivery System for Breast Cancer Therapy. Int. J. Nanomed..

[141] Wei Y.M., Liang J., Zheng X.L., Pi C., Liu H., Yang H.R., Zou Y.G., Ye Y., Zhao L. (2017). Lung-targeting drug delivery system of baicalin-loaded nanoliposomes: Development, biodistribution in rabbits, and pharmacodynamics in nude mice bearing orthotopic human lung cancer. Int. J. Nanomed..

[142] Xu Q., Zhou A., Wu H.F., Bi Y.J. (2019). Development and in vivo evaluation of baicalin-loaded W/O nanoemulsion for lymphatic absorption. Pharm. Dev. Technol..

[143] Wang X., Lu J., Cao Y., Liang Y.D., Dai X.L., Liu K., Xie L., Li X.F. (2023). Does binary blend emulsifier enhance emulsifier performance? Preparation of baicalin nanoemulsions using tea saponins and glycyrrhizic acid as binary blend emulsifier. J. Drug Deliv. Sci. Technol..

[144] Memariani Z., Abbas S.Q., Ul Hassan S.S., Ahmadi A., Chabra A. (2021). Naringin and naringenin as anticancer agents and adjuvants in cancer combination therapy: Efficacy and molecular mechanisms of action, a comprehensive narrative review. Pharmacol. Res..

[145] Fernández J., Silván B., Entrialgo-Cadierno R., Villar C.J., Capasso R., Uranga J.A., Lombó F., Abalo R. (2021). Antiproliferative and palliative activity of flavonoids in colorectal cancer. Biomed. Pharmacother..

[146] Bhia M., Motallebi M., Abadi B., Zarepour A., Pereira-Silva M., Saremnejad F., Santos A.C., Zarrabi A., Melero A., Jafari S.M. (2021). Naringenin Nano-Delivery Systems and Their Therapeutic Applications. Pharmaceutics.

[147] Slika H., Mansour H., Wehbe N., Nasser S.A., Iratni R., Nasrallah G., Shaito A., Ghaddar T., Kobeissy F., Eid A.H. (2022). Therapeutic potential of flavonoids in cancer: ROS-mediated mechanisms. Biomed. Pharmacother..

[148] Lesjak M., Beara I., Simin N., Pintac D., Majkic T., Bekvalac K., Orcic D., Mimica-Dukic N. (2018). Antioxidant and anti-inflammatory activities of quercetin and its derivatives. J. Funct. Foods.

[149] Wang Z.X., Ma J., Li X.Y., Wu Y., Shi H., Chen Y., Lu G., Shen H.M., Lu G.D., Zhou J. (2021). Quercetin induces p53-independent cancer cell death through lysosome activation by the transcription factor EB and Reactive Oxygen Species-dependent ferroptosis. Br. J. Pharmacol..

[150] Hemati M., Haghiralsadat F., Yazdian F., Jafari F., Moradi A., Malekpour-Dehkordi Z. (2019). Development and characterization of a novel cationic PEGylated niosome-encapsulated forms of doxorubicin, quercetin and siRNA for the treatment of cancer by using combination therapy. Artif. Cells Nanomed. Biotechnol..

[151] Karole A., Parvez S., Thakur R.S., Mudavath S.L. (2022). Effervescent based nano-gas carrier enhanced the bioavailability of poorly aqueous soluble drug: A comprehensive mechanistic understanding. J. Drug Deliv. Sci. Technol..

[152] Lee J.H., Yeo Y. (2015). Controlled drug release from pharmaceutical nanocarriers. Chem. Eng. Sci..

[153] Herdiana Y., Wathoni N., Shamsuddin S., Muchtaridi M. (2022). Drug release study of the chitosan-based nanoparticles. Heliyon.

[154] Kashkooli F.M., Soltani M., Souri M. (2020). Controlled anti-cancer drug release through advanced nano-drug delivery systems: Static and dynamic targeting strategies. J. Control. Release.

[155] Borandeh S., van Bochove B., Teotia A., Seppälä J. (2021). Polymeric drug delivery systems by additive manufacturing. Adv. Drug Deliv. Rev..

[156] Kamaly N., Yameen B., Wu J., Farokhzad O.C. (2016). Degradable Controlled-Release Polymers and Polymeric Nanoparticles: Mechanisms of Controlling Drug Release. Chem. Rev..

[157] Huh K.M., Kang H.C., Lee Y.J., Bae Y.H. (2012). pH-sensitive polymers for drug delivery. Macromol. Res..

[158] Anirudhan T.S., Manjusha V., Sekhar V.C. (2021). A new biodegradable nano cellulose-based drug delivery system for pH-controlled delivery of curcumin. Int. J. Biol. Macromol..

[159] Gong X., Qiu X., Zhao Y., Wang J. (2022). Synthesis and characterization of pH responsive tea polyphenols/mesoporous zinc oxide nano-complex with antioxidant and antibacterial activity. J. Funct. Mater..

[160] Weinstain R., Slanina T., Kand D., Klán P. (2020). Visible-to-NIR-Light Activated Release: From Small Molecules to Nanomaterials. Chem. Rev..

[161] Zhou Z.X., Vázquez-González M., Willner I. (2021). Stimuli-responsive metal-organic framework nanoparticles for controlled drug delivery and medical applications. Chem. Soc. Rev..

[162] Qian C.G., Yu J.C., Chen Y.L., Hu Q.Y., Xiao X.Z., Sun W.J., Wang C., Feng P.J., Shen Q.D., Gu Z. (2016). Light-Activated Hypoxia-Responsive Nanocarriers for Enhanced Anticancer Therapy. Adv. Mater..

[163] Hu D., Zhang C., Sun C., Bai H., Xie J., Gu Y., Li M., Jiang J., Le A., Qiu J. (2023). Carvacrol combined with NIR light-responsive nano-drug delivery system with specific anti-bacteria anti-inflammation, and immunomodulation for periodontitis. Nano Res..

[164] Wen X., Liu N., Ren J., Jiao X., Lv J., Akhtar M.H., Qi H., Zhu J., Yu C., Li Y. (2022). In situ synthesis of a functional ZIF-8 nanocomposite for synergistic photodynamic-chemotherapy and pH and NIR-stimulated drug release. New J. Chem..

[165] Meerovich I., Nichols M.G., Dash A.K. (2019). Low-intensity light-induced paclitaxel release from lipid-based nano-delivery systems. J. Drug Target..

[166] Li F.Y., Pei Z.R., Chen S.T., Li G., Liu M.Y., Ding L.Q., Liu J.B., Qiu F. (2024). Multifunctional nano-herb based on tumor microenvironment for enhanced tumor therapy of gambogic acid. Chin. Chem. Lett..

[167] Hafezi M., Rostami M., Hosseini A., Rahimi-Nasrabadi M., Fasihi-Ramandi M., Badiei A., Ahmadi F. (2021). Cur-loaded ZnFe_2_O_4_@mZnO@N-GQDs biocompatible nano-carriers for smart and controlled targeted drug delivery with pH-triggered and ultrasound irradiation. J. Mol. Liq..

[168] Xu H.Y., Zhang Y.Q., Liu Z.M., Chen T., Lv C.Y., Tang S.H., Zhang X.B., Zhang W., Li Z.Y., Zhou R.R. (2019). ETCM: An encyclopaedia of traditional Chinese medicine. Nucleic Acids Res..

[169] Kou L.F., Bhutia Y.D., Yao Q., He Z.G., Sun J., Ganapathy V. (2018). Transporter-Guided Delivery of Nanoparticles to Improve Drug Permeation across Cellular Barriers and Drug Exposure to Selective Cell Types. Front. Pharmacol..

[170] Shi S.Z., Zhong H.B., Zhang Y., Mei Q.S. (2024). Targeted delivery of nano-radiosensitizers for tumor radiotherapy. Coord. Chem. Rev..

[171] Duan L., Yang L., Jin J., Yang F., Liu D., Hu K., Wang Q.X., Yue Y.B., Gu N. (2020). Micro/nano-bubble-assisted ultrasound to enhance the EPR effect and potential theranostic applications. Theranostics.

[172] Cheng X.X., Xie Q.R., Sun Y. (2023). Advances in nanomaterial-based targeted drug delivery systems. Front. Bioeng. Biotechnol..

[173] Ren H.W., He Y.W., Liang J.M., Cheng Z.K., Zhang M., Zhu Y., Hong C., Qin J., Xu X.C., Wang J.X. (2019). Role of Liposome Size, Surface Charge, and PEGylation on Rheumatoid Arthritis Targeting Therapy. ACS Appl. Mater. Interfaces.

[174] Meng Q.W., Li J.W., Wang C.S., Shan A.S. (2023). Biological function of resveratrol and its application in animal production: A review. J. Anim. Sci. Biotechnol..

[175] Tian B.R., Liu J.Y. (2020). Resveratrol: A review of plant sources, synthesis, stability, modification and food application. J. Sci. Food Agric..

[176] Vijayakumar M.R., Kumari L., Patel K.K., Vuddanda P.R., Vajanthri K.Y., Mahto S.K., Singh S. (2016). Intravenous administration of *trans*-resveratrol-loaded TPGS-coated solid lipid nanoparticles for prolonged systemic circulation, passive brain targeting and improved in vitro cytotoxicity against C6 glioma cell lines. RSC Adv..

[177] Fang X.L., Cao J.J., Shen A.Z. (2020). Advances in anti-breast cancer drugs and the application of nano-drug delivery systems in breast cancer therapy. J. Drug Deliv. Sci. Technol..

[178] Wang M.J., Xue W.X., Yuan H.H., Wang Z.C., Yu L. (2024). Nano-Drug Delivery Systems Targeting CAFs: A Promising Treatment for Pancreatic Cancer. Int. J. Nanomed..

[179] Tu Z.X., Zhong Y.L., Hu H.Z., Shao D., Haag R.N., Schirner M., Lee J., Sullenger B., Leong K.W. (2022). Design of therapeutic biomaterials to control inflammation. Nat. Rev. Mater..

[180] Takahashi T., Utoguchi N., Takara A., Yamamoto N., Nakanishi T., Tanaka K., Audus K.L., Watanabe Y. (2001). Carrier-mediated transport of folic acid in BeWo cell monolayers as a model of the human trophoblast. Placenta.

[181] Roger E., Kalscheuer S., Kirtane A., Guru B.R., Grill A.E., Whittum-Hudson J., Panyam J. (2012). Folic Acid Functionalized Nanoparticles for Enhanced Oral Drug Delivery. Mol. Pharm..

[182] Tong L.X., Chen W., Wu J., Li H.X. (2014). Folic acid-coupled nano-paclitaxel liposome reverses drug resistance in SKOV3/TAX ovarian cancer cells. Anti-Cancer Drugs.

[183] Du Z.J., Mao Y., Zhang P.F., Hu J., Fu J.J., You Q.J., Yin J. (2021). TPGS-Galactose-Modified Polydopamine Co-delivery Nanoparticles of Nitric Oxide Donor and Doxorubicin for Targeted Chemo-Photothermal Therapy against Drug-Resistant Hepatocellular Carcinoma. ACS Appl. Mater. Interfaces.

[184] Chen Q.B., Li Q., Liang Y.Q., Zu M.H., Chen N.X., Canup B.S.B., Luo L.Y., Wang C.H., Zeng L., Xiao B. (2022). Natural exosome-like nanovesicles from edible tea flowers suppress metastatic breast cancer via ROS generation and microbiota modulation. Acta Pharm. Sin. B.

[185] Nie W.D., Wu G.H., Zhang J.F., Huang L.L., Ding J.J., Jiang A.Q., Zhang Y.H., Liu Y.H., Li J.C., Pu K.Y. (2020). Responsive Exosome Nano-bioconjugates for Synergistic Cancer Therapy. Angew. Chem.-Int. Ed..

[186] Wang H.H., Wang B.Y., Wang S.S., Chen J.Q., Zhi W.W., Guan Y.B., Cai B.R., Zhu Y.H., Jia Y.Y., Huang S.N. (2022). Injectable in situ intelligent thermo-responsive hydrogel with glycyrrhetinic acid-conjugated nano graphene oxide for chemo-photothermal therapy of malignant hepatocellular tumor. J. Biomater. Appl..

[187] Iskusnykh I.Y., Zakharova A.A., Pathak D. (2022). Glutathione in Brain Disorders and Aging. Molecules.

[188] Zhan Y., Qiu Y., Wang H., Wang Z., Xu J., Fan G., Xu J., Li W., Cao Y., Le V.-M. (2020). Bufalin reverses multidrug resistance by regulating stemness through the CD133/nuclear factor-κB/MDR1 pathway in colorectal cancer. Cancer Sci..

[189] Yang S.D., Zhu W.J., Zhu Q.L., Chen W.L., Ren Z.X., Li F., Yuan Z.Q., Li J.Z., Liu Y., Zhou X.F. (2017). Binary-copolymer system base on low-density lipoprotein-coupled N-succinyl chitosan lipoic acid micelles for co-delivery MDR1 siRNA and paclitaxel, enhances antitumor effects via reducing drug. J. Biomed. Mater. Res. Part B Appl. Biomater..

[190] Ouyang L., Wang L.S., Schork F.J. (2011). Synthesis and nucleation mechanism of inverse emulsion polymerization of acrylamide by RAFT polymerization: A comparative study. Polymer.

[191] Maeki M., Kimura N., Sato Y., Harashima H., Tokeshi M. (2018). Advances in microfluidics for lipid nanoparticles and extracellular vesicles and applications in drug delivery systems. Adv. Drug Deliv. Rev..

[192] Cooley M., Sarode A., Hoore M., Fedosov D.A., Mitragotri S., Sen Gupta A. (2018). Influence of particle size and shape on their margination and wall-adhesion: Implications in drug delivery vehicle design across nano-to-micro scale. Nanoscale.

[193] Zhang T.H., Wang L.S., He X.Y., Lu H.L., Gao L. (2022). Cytocompatibility of pH-sensitive, chitosan-coated Fe_3_O_4_ nanoparticles in gynecological cells. Front. Med..

[194] Yin M.Z., Xu W.G., Cui B.C., Dai H.L., Han Y.C., Yin Y.X., Li S.P. (2014). Effects of the Interaction between Hydroxyapatite Nanoparticles and Hepatoma Cells. J. Wuhan Univ. Technol.-Mater. Sci. Ed..

[195] Kim J.E., Kim H., An S.S.A., Maeng E.H., Kim M.K., Song Y.J. (2014). In vitro cytotoxicity of SiO_2_ or ZnO nanoparticles with different sizes and surface charges on U373MG human glioblastoma cells. Int. J. Nanomed..

[196] Tay C.Y., Cai P.Q., Setyawati M.I., Fang W.R., Tan L.P., Hong C.H.L., Chen X.D., Leong D.T. (2014). Nanoparticles Strengthen Intracellular Tension and Retard Cellular Migration. Nano Lett..

