# Correction: Chen et al. Enhancing the Efficacy of Active Pharmaceutical Ingredients in Medicinal Plants through Nanoformulations: A Promising Field. *Nanomaterials* 2024, *14*, 1598

**DOI:** 10.3390/nano15181442

**Published:** 2025-09-19

**Authors:** Yuhao Chen, Yuying Tang, Yuanbo Li, Yukui Rui, Peng Zhang

**Affiliations:** 1 Beijing Key Laboratory of Farmland Soil Pollution Prevention and Remediation, College of Resources and Environmental Sciences, China Agricultural University, Beijing 100093, China; 18855380430@163.com (Y.C.); liyuanbo@cau.edu.cn (Y.L.); 2 Tangshan Jinhai New Material Co., Ltd., Tangshan 063000, China; 3 Faculty of Resources and Environment, China Agricultural University, Shanghe County Baiqiao Town Science and Technology Courtyard, Jinan 250100, China; 4 Department of Environmental Science and Engineering, University of Science and Technology of China, Hefei 230026, China

In the original publication [1], reference [189], “Guo, H.C.; Liu, F.; Yang, S.G.; Xue, T. Emodin alleviates gemcitabine resistance in pancreatic cancer by inhibiting MDR1/P-glycoprotein and MRPs expression. *Oncol. Lett.* **2020**, *20*, 167” was retracted, and the authors would like to delete it. With this correction, the order of some references has been adjusted accordingly. The authors state that the scientific conclusions are unaffected. This correction was approved by the Academic Editor. The original publication has also been updated.
